# Microbial communities across activated sludge plants show recurring species-level seasonal patterns

**DOI:** 10.1038/s43705-022-00098-4

**Published:** 2022-02-18

**Authors:** Miriam Peces, Giulia Dottorini, Marta Nierychlo, Kasper Skytte Andersen, Morten Kam Dahl Dueholm, Per Halkjær Nielsen

**Affiliations:** Department of Chemistry and Bioscience, Section of Biotechnology, Center for Microbial Communities, Aalborg University, Aalborg East, 9220 Denmark

**Keywords:** Environmental sciences, Water microbiology

## Abstract

Microbial communities in activated sludge (AS) are the core of sanitation in wastewater treatment plants (WWTPs). Microbial communities in AS have shown seasonal changes, however, long-term experiments (>2 years) are rarely conducted, limiting our understanding of the true seasonal dynamics in WWTPs. In this study, we resolved the microbial seasonal dynamics at the species level in four municipal full-scale WWTPs, sampled every 7–10 days, during 3–5 consecutive years. By applying a new time-series analysis approach, we revealed that the seasonal pattern was species-specific, where species belonging to the same functional guild or genus may show different seasonal dynamics. Species could be grouped into cohorts according to their seasonal patterns, where seasonal cohorts showed repeatable annual dynamics across years and plants. Species were also grouped according to their net growth rate in the AS (i.e., growing species and disappearing species). Growing species were more prevailing in spring and autumn cohorts, while disappearing species, which were only present due to the continuous immigration from influent wastewater, were mostly associated with winter and spring cohorts. Most known process-critical species, such as nitrifiers, polyphosphate accumulating organisms and filamentous organisms, showed distinct species-specific patterns. Overall, our study showed that overarching seasonal patterns affected microbial species in full-scale AS plants, with similar seasonal patterns across plants for many dominant species. These recurrent seasonal variations should be taken into account in the operation, understanding and management of the WWTPs.

## Introduction

Microbial communities in activated sludge (AS) are the core of wastewater treatment plants (WWTPs) worldwide, where organic pollutants and nutrients are transformed by the action of diverse microbial groups to produce clean water, and in more advanced configurations, recover resources such as phosphorus. The understanding of the microbial communities in WWTPs is being continuously resolved and improving [1], and a good understanding of the factors determining AS community assembly and dynamics is important for informed management of the WWTPs. The microbial community assembly is determined by a variety of factors, with the relative importance of different factors varying across WWTPs with different process design and operation. The controlling factors can be deterministic, such as environmental factors (e.g., wastewater temperature, the chemical composition of the influent wastewater), process design, operation (e.g., solid retention time (SRT), aeration time, chemicals dosage [2, 3]), and neutral, such as dispersal (i.e., microbial immigration from influent wastewater) [4, 5]. Given the multiple factors affecting the AS, it is clear that the composition and assembly of the microbial community are exposed to a variety of temporal responses that can alter its dynamics. Indeed, the few published longitudinal AS studies with good temporal resolution (e.g., ≥1 sample/month) have shown seasonal variations in community composition and abundance over the calendar year in different climate zones [6–13].

The degree to which the seasonal variations of the microbial communities in AS are cyclic is not well documented. Most longitudinal studies have been carried out only during one year, so it is unknown whether the seasonal variation is repeatable over the years, indicating a seasonal periodicity, or if the changes only occurred over a few months. In a 2-year survey, Flowers et al. [8] observed a repeatable seasonal pattern in microbial richness and diversity, whereas in a 5-year survey by Ju and Zhang [14], the overall community composition appeared seasonally independent. Although two studies are not enough to determine if seasonal periodicity is WWTP-specific, a key observation from these studies is the presence of different dynamic responses for individual taxa. For example, in Ju and Zhang [14] a *Tetrasphaera*-related operational taxonomic unit (OTU), clustered at 97% sequence identity, had a clear seasonal periodicity, while a *Nitrosomonas*-related OTU showed random fluctuations. Additionally, 1-year surveys have also shown the presence, and absence, of temporal variations for process-critical taxa [9, 10, 15, 16]. However, it is currently not known if some overarching factors are driving these variations and whether they are taxa-specific and/or WWTP-specific.

A great challenge interpreting seasonal variation in previous studies was the lack of species-level taxonomic classification. Previous studies aggregated taxa (usually resolved as 16S rRNA gene OTUs) in genera or families, or into functional guilds such as nitrifiers, polyphosphate accumulating organisms (PAOs) or filamentous organisms (hereafter referred to as filaments). This is problematic since not all species in the same guild may share the same ecophysiological traits. Consequently, grouping species could mask any species-level dynamics. This problem can be solved by using ecosystem-specific reference databases which can resolve the taxonomy to species level (>98.7% sequence identity) [17, 18]. For WWTPs and anaerobic digesters, the improved ecosystem-specific reference database MiDAS 4 provides reproducible species-level classifications based on a comprehensive set of amplicon sequence variant (ASV) resolved full-length 16S rRNA gene reference sequences. Moreover, it provides placeholder names for unclassified environmental taxa, providing a common taxonomy for all studies in the field [1]. This approach has shown that many dominant species are shared among WWTPs with similar process configuration [18]. Therefore, by comparing several WWTPs, it may be possible to find species-specific recurrent patterns.

Resolving species dynamics is important since many metabolic and functional traits are only conserved at the highest taxonomic resolution, which allows assigning known and putative functional roles to individual microorganisms [17–19]. A recent example to illustrate the importance of species-level resolution is the putative foam-forming genus *Candidatus* Microthrix [20]. In Danish WWTPs, *Ca*. M. parvicella and the novel *Ca*. M. subdominans (previous MiDAS 3 placeholder species name midas_s_2) are the two main coexisting species, and they show very different dynamics. Both species had substantial effects on the sludge settling properties, but *Ca*. M. parvicella showed a strong seasonality proliferating in the coldest months, while *Ca*. M. subdominans did not show any seasonal pattern [20]. Additionally, the combination of reproducible species-level classification and biomass mass-balances including immigration from influent wastewater, allows the grouping of species according to their net growth rate in the AS [4, 21]. Briefly, ‘growing species’ are expected to grow in the AS and perform some critical process functions, while ‘disappearing species’ are expected to die-off in the AS and are only present because they are constantly transported with the influent wastewater [4]. The study of these growth groups separately can improve our understanding of the microbial community assembly since the growing species may be very dependent on temperature and process operation, while the disappearing species mainly depend on the immigration from the sewer system.

In this study, we investigated the seasonal periodicity of all species in four full-scale nutrient removal WWTPs in temperate climate during a longitudinal survey of 3–5 consecutive years. The aims were to evaluate (i) if the microbial community structure could be seasonally described, (ii) which species showed significant seasonal variations and if their dynamics were the same across different WWTPs, (iii) if similar seasonal patterns were observed for species within genera and functional guilds, and (iv) if growing species showed similar temporal dynamics to disappearing species, since the latter are present only due to transportation by the influent wastewater.

## Materials and methods

### Wastewater treatment plant characterisation and sample collection

This longitudinal survey was conducted between 2015 and 2020 in four full-scale municipal WWTPs operated as conventional AS with nitrogen and enhanced biological phosphorus removal (EBPR) configuration. The four plants, Aalborg W, Aalborg E, Damhusåen and Randers, ran without major disturbances and operational changes during the sampling period. Only sporadic bulking events were reported by plant operators, with effluent concentrations consistently complying with the Danish effluent discharge limits (BOD_5_ < 15 mg/L, total nitrogen <8 mg/L, and total phosphorus < 1.5 mg/L) [22, 23]. The four plants are medium size municipal WWTPs (130 000 to 350 000 PE) with average SRTs of 10–30 days and a yearly temperature range of 7–20 °C. The plants had minor differences in design configuration, operational conditions, and influent composition (mostly municipal sewage but some discharge from nearby industries) that made each plant unique from an operational perspective (Supplementary Table S1).

AS samples were collected from the aeration tanks, or at the end of the aeration phase for plants with alternating operation, every 7–10 days between 2015 and 2020 (~1000 samples, Table S1). Routine monitoring at Damhusåen (line B) started in 2017. Briefly, 500 mL of AS were collected, homogenised and subsampled in 2 mL cryotubes as detailed in the MiDAS field guide (https://www.midasfieldguide.org/guide/protocols). The samples were immediately stored at −18 °C at the WWTPs and shipped frozen to our lab in batches. Samples were stored at − 18 °C until further processing.

### Amplicon sequencing

Detailed sample preparation, DNA extraction and purification protocol used can be found in the MiDAS field guide (https://www.midasfieldguide.org/guide/protocols). Concisely, 160 μL of a homogeneous sample was used for DNA extraction with the FastDNA® spin kit for soil (MP biomedicals) and FastPrep-96 bead beater (MP Biomedicals) following the manufacturer’s protocol with minor modifications in the bead beating intensity and purification. The V1–V3 region of 16S rRNA gene was amplified using the 27F (3′-AGAGTTTGATCCTGGCTCAG-5′) [24] and 534R (3′-ATTACCGCGGCTGCTGG-5′) [25] primers, as this primer set has shown to give the most representative community structure and the highest taxonomic resolution for bacteria in AS systems [17, 26]. Amplicon sequencing was conducted using the Illumina MiSeq platform (Illumina, USA) as described in Dottorini et al. [4].

Amplicon sequencing data were processed with AmpProc v.5.1.0 for downstream analyses (https://github.com/eyashiro/AmpProc). Briefly, only forward sequencing reads were processed using usearch v.11.0.667 [27]. Raw fastq files were filtered for phiX sequences using usearch -filter_- phix, trimmed to 250 bp using usearch -fastx_truncate –trunclen 250, and quality filtered using usearch -fastq_filter with -fastq_- maxee 1.0. The sequences were dereplicated using usearch -fastx_uniques with -sizeout. ASVs were generated using -unoise3 [28] with standard settings. The ASVs were mapped to the full-length ASVs (FL-ASVs) of the MiDAS 4 wastewater ecosystem-specific reference database, allowing species resolution [1, 17] (available at https://www.midasfieldguide.org/guide/downloads). Taxonomy was assigned using the SINTAX classifier with a confidence threshold of 0.8 [29]. In the MiDAS 4, taxonomic names are based on reproducible clustering with rank-specific identity thresholds and placeholder names [1]. Species-level classification (>98.7% sequence identity as recommended by Yarza et al. [30]) was chosen to improve the read counts available for each investigated taxon and to provide a reproducible name recognisable across studies. For ASVs without species-level classification, the taxonomy was assigned at the lowest available taxonomic level (e.g., genus), and these ASVs were treated as separate species. Microbial species were assigned to known functional guilds (e.g., nitrifiers, PAO) based on the main in situ metabolism described to occur in AS (https://www.midasfieldguide.org/guide/search).

### Data analysis

#### Downstream analyses and visualisation

Downstream statistical analyses and visualisation were performed in R v.4.0.3 [31] mainly using the following packages: tidyverse v.1.3.0 [32], ampvis2 v.2.6.1 [33] vegan v.2.5.6 [34], ComplexUpset v.1.1.0 [35], ggseas v.0.5.4 [36], and Harmonic Regression v.1.0 [37]. Prior to data analysis, samples with less than 10 000 reads were discarded and duplicate samples from the same sampling point were combined by averaging the relative abundance of each ASV. The total reads per sample ranged from 10 893 to 170 639 (Fig. S1). For alpha-diversity analyses, samples were rarefied at 10 000 reads and the number of unique ASVs and Simpson index were calculated using ampvis2.

Differences in overall microbial community structure were explored by principal component analysis (PCA), where the ASV reads were Hellinger transformed prior to ordination using ampvis2. Statistical differences between PCA clusters were assessed by PERMANOVA using the adonis2 function in the vegan R package. The relative abundance of species was visualised with boxplots using the mean relative abundance for each plant.

Prior to time-series analyses (described in “Time-series decomposition and grouping in seasonal cohorts”) sequencing reads were transformed using robust-centred log-ratio (rclr) transformation to account for the compositional and sparse nature of the data as in Martino et al. [38]:

1 $${{{{{{{\mathrm{rclr}}}}}}}}\left( {{{{x}}}_{{{i}}}} \right) = \log \left[ {\frac{{{{{g}}}\left( {{{{x}}}_{{{i}}}} \right)}}{{{{{{{{{\mathrm{geometric}}}}}}}}\,{{{{{{{\mathrm{mean}}}}}}}}\left( {{{x}}} \right)}}} \right]$$

2 $${{{{{{{\mathrm{geometric}}}}}}}}\,{{{{{{{\mathrm{mean}}}}}}}}\left( {{{x}}} \right) = \left( {\mathop {\prod}\nolimits_{{{{i}}} = 1}^{{{N}}} {{{{{{{{\mathrm{g}}}}}}}}({{{x}}}_{{{i}}})} } \right)^{\frac{1}{{{{N}}}}}$$

The rclr transformation is based on the log-ratio transformation introduced by Aitchison [39] where the rclr is the logarithm after dividing the number of reads of each species (*g*(*x*_*i*_)) by the geometric mean of the total sample reads (geometric mean(*x*)) for taxa (*N*) with a read abundance >0.

#### Time-series decomposition and grouping in seasonal cohorts

For each WWTP, only species with a relative abundance >0.05% in at least one sample were retained to find recurrent seasonal variations. To decrease the dimensionality inherently contained in time-series data [40], each species time-series was decomposed into trend, season and residual components based on local smoothing regression [41]. The time-series components were extracted using the function stl() implemented in the ggseas R package with an s.window = “periodic” and frequency = 52. Before time-series decomposition, species reads were rclr transformed (section “Downstream analyses and visualisation”). When required, linear interpolation between dates was used to create an even weekly sampling distribution across years. The extracted seasonal component was used for further data processing and analyses. Each species seasonal component was fitted to a simple harmonic model (Eq. (3)) to determine the statistical significance of the seasonal response (1% significance threshold, *p* < 0.01):

3 $${{{y}}} = {{{m}}} + {{{A}}} \cdot {{{{{{{\mathrm{cos}}}}}}}}\left( {\omega \frac{{{{t}}}}{{2\pi }} - \varphi } \right)$$

where *m* is the mean value of the seasonal component, *A* is the amplitude of the oscillation, *ω* is the frequency of the oscillation, *t* is time (in days) and *φ* is the phase of the oscillation. A visual summary of this approach can be found in Supplementary Fig. S2.

Species were assigned into seasonal cohorts depending on the temporal location of the maximum seasonal peak. The seasonal cohorts were based on the yearly process tank temperature variation (Fig. S3), using as a reference the definition of astronomical seasons for the northern hemisphere:

- Winter cohort: Species that have the maximum peak located between the 21st December and 20th March. Process tank temperature is about 10–12 °C.
- Spring cohort: Species that have the maximum peak located between the 21st March and the 21st June. Process tank temperature increases from about 11 to approximately 17 °C.
- Summer cohort: Species that have the maximum peak located between the 22nd June and the 22nd September. Process tank temperature is about 17–19 °C.
- Autumn cohort: Species that have the maximum peak located between the 23rd September and the 22nd December. Process tank temperature decreases from about 17 to approximately 12 °C.
- Non-significant cohort: Species with a non-significant harmonic model fit (*p* > 0.01).

The strength of the seasonal component was calculated based on the variance explained by the seasonal component over the residual component (Eq. (4)) [42].

4 $${{{{{{{\mathrm{Fs}}}}}}}} = 1 - \frac{{{{{{{{{\mathrm{variance}}}}}}}}({{{{{{{\mathrm{residual}}}}}}}}\,{{{{{{{\mathrm{component}}}}}}}})}}{{{{{{{{{\mathrm{variance}}}}}}}}\left( {{{{{{{{\mathrm{residual}}}}}}}}\,{{{{{{{\mathrm{component}}}}}}}}} \right) + {{{{{{{\mathrm{variance}}}}}}}}({{{{{{{\mathrm{seasonal}}}}}}}}\,{{{{{{{\mathrm{component}}}}}}}})}}$$

Fs takes values from 0 to 1, 1 indicating that variation in species abundance is completely explained by the seasonal variation. For species with a significant seasonal component (*p* < 0.01, harmonic model fit), we defined 5 categories for seasonal strength based on the distribution of Fs values obtained in this study (Fig. S4): Fs > 0.75, Very strong; 0.75 > Fs > 0.55, strong; 0.55 > Fs > 0.35, Moderate; 0.35 > Fs > 0.15, Weak; Fs < 0.15, Very weak.

#### Classification of activated sludge bacteria into growth groups

Most species in AS are detected in the influent wastewater, therefore it is possible to identify whether those species grow or die in AS based on mass balances [4]. In this study, we assigned the species according to their growth fate as identified by Dottorini et al. [4] since the WWTPs in this study were present among the WWTPs studied by Dottorini et al. [4], except for Damhusåen. However, Damhusåen WWTP has very similar taxa, process design and geographical location to our previous dataset, therefore it can be assumed that species will follow the same fate in AS. Species in WWTPs with different process design may not follow the same growth fate. The detailed mass-balance methodology can be found in Dottorini et al. [4]. Briefly, paired samples from influent wastewater and AS were collected from 11 municipal WWTPs across Denmark every second week for months. The net growth rate of bacterial species in the AS process was estimated based on a mass-balance between paired influent and AS samples, knowing the flow-rates of the plant and assuming: (i) steady-state process, (ii) that the apparent net growth rate (*k*) can be described as a first-order process and (iii) that the biomass concentration of a species can be described by the relative abundance of that species multiplied by the total number of cells. According to the mass-balance, species can be assigned to three different groups:

- Growing, where the apparent net growth rate is positive (*k* > 0).
- Disappearing, where the apparent net growth rate is negative (k < 0).
- Surviving, where the apparent net growth rate is close to zero (*k* ⋍ 0) and they may slowly grow or disappear depending on the process conditions.

Species that could not be unequivocally assigned to any of the growth groups across WWTPs or species with a relative read abundance below 0.05% were assigned to the ambiguous group. It was considered that the low number of reads for species with a relative read abundance below 0.05% contains too much uncertainty to be thoroughly classified. The detailed methodology can be found in Dottorini et al. [4].

## Results

### Microbial composition and time-series dynamics of growth groups in full-scale WWTPs

**Fig. 1:**
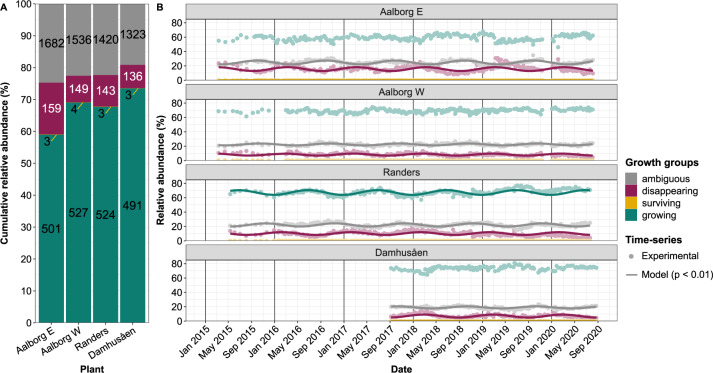
Distribution of growth groups in four WWTPs over time. **A** Mean cumulative relative abundance of growth groups per sample. Values in the bars show the number of different species in each fraction. **B** Time-series of cumulative read abundance of each growth group, where dots show the experimental results and the solid lines show the corresponding harmonic model fits. Only significant (*p* < 0.01) harmonic fits are shown.

Species were classified according to their growth group (i.e., growing, disappearing, surviving or ambiguous) to evaluate: (i) the relative contribution of each growth group to the total read abundance, (ii) the identity and relative read abundance of species in each growth group, and (iii) their temporal dynamics in each WWTP. The growing fraction was dominant in all plants, representing about 60–70% of the total relative read abundance, and consisting of about 500 different species. The disappearing group contributed 8–15% of the relative read abundance in each WWTP, consisting of about 150 species (Fig. 1A). This group was only present due to the continuous immigration with the influent wastewater. Very few surviving species were sparsely observed in low abundance during the studied period. The ambiguous fraction was large, it harboured 1300–1600 species found in very low relative abundance, corresponding to a cumulative total read abundance of 20–25% in each WWTP (Fig. 1A). The growth groups showed a distinct dynamic response over time (Fig. 1B). The growing fraction showed significant yearly seasonal dynamics only in one plant (Randers), whereas the disappearing and ambiguous fractions showed consistent yearly seasonal dynamics in all plants with maximum abundance around February–March and September–October, respectively.

**Fig. 2:**
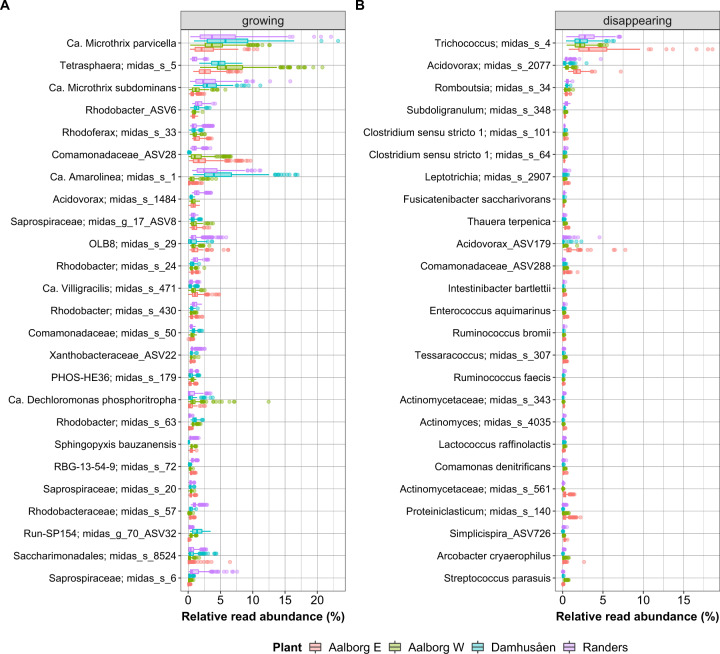
Relative read abundance of the top 25 most abundant bacterial species in the four WWTPs. **A** Species in the growing group, **B** species in the disappearing group. The species in the ambiguous and surviving group are shown in Fig. S5.

Within the most abundant bacteria in the growing fraction (Fig. 2), we found species and genera typical for Danish and global EBPR plants [1, 18] such as filamentous *Ca*. Microthrix and *Ca*. Amarolinea, the PAO *Tetrasphaera* and *Dechloromonas*, and other genera with unknown or poorly described in situ functions such as *Rhodobacter*, *Rhodoferax*, OLB8 and midas_g_17 (both family *Saprospiraceae*). Nitrifiers were mostly represented by *Nitrosomonas* (midas_s_139 and midas_s_717), *Nitrotoga* (midas_s_181 and ASV223), and *Nitrospira defluvii*, ranking within the top 100 most abundant growing species. Additionally, the most abundant growing bacteria were more evenly distributed than the disappearing species, which were dominated by *Trichoccocus* midas_s_4. The most abundant species of the ambiguous and surviving fraction are shown in Supplementary Fig. S5.

### Microbial community structure can be seasonally described

The mean number of ASVs per sample was 774 for the growing fraction and 335 for the disappearing fraction, and the mean Simpson index (reported as 1-D) was slightly higher for the growing fraction (0.985) compared to the disappearing fraction (0.958), confirming that growing communities were more diverse than the disappearing fraction (Fig. 2).

To evaluate the yearly dynamics of the community structure, alpha-diversity was calculated for each plant and fitted to a simple harmonic model (Eq. 1) (Fig. S6). For most plants and diversity estimates, the model fit was significant (*p* < 0.01) indicating a seasonal response. Damhusåen was an exception, where the seasonality of the number of disappearing ASVs was not significant (*p* = 0.5097) and for the growing species, Simpson diversity was above the 99% confidence threshold (*p* = 0.0272). However, Damhusåen was the plant with the least sampling years. Overall, both diversity estimates showed a similar seasonal yearly pattern for all the plants, with a minimum diversity in late winter to early spring, and maximum richness in late summer to early autumn, for both growing and disappearing species groups.

**Fig. 3:**
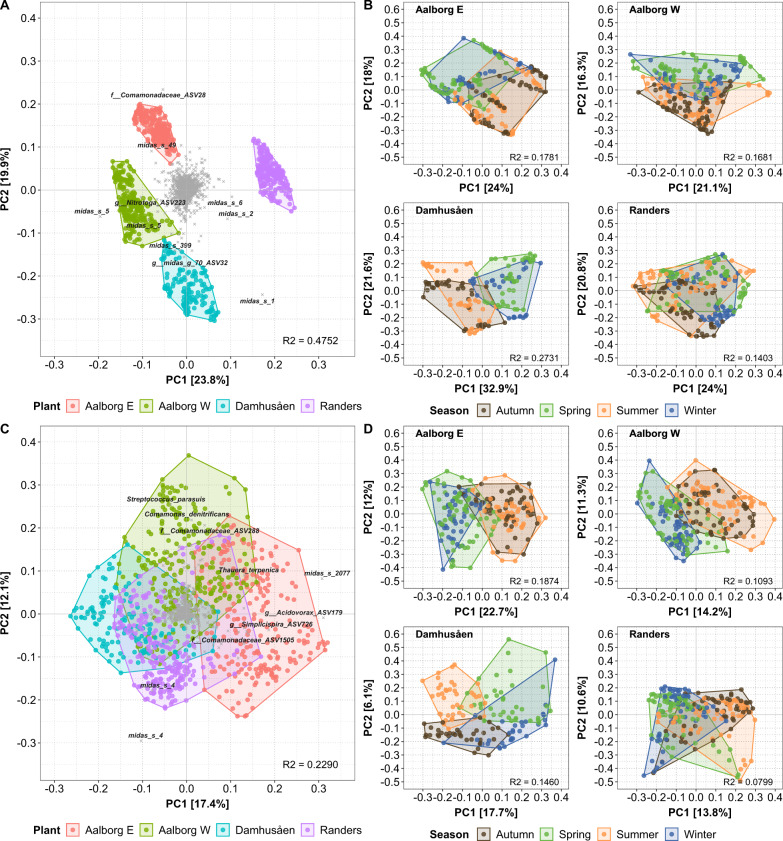
PCA plots of the bacterial community structure. **A** Differences across plants for growing bacteria, **B** seasonal differences for growing bacteria in each plant, **C** differences across plants for disappearing bacteria, **D** seasonal differences for disappearing bacteria in each plant. R^2^ values show the explained variance by the grouping variable (**A**, **C**: WWTP; **B**, **D**: Season).

The growing communities in the four WWTPs showed distinct clustering in PCA analysis (Fig. 3A) with a different community structure depending on the composition and abundance of each ASV, as also indicated at species level (Fig. 2). The disappearing communities also showed some plant-specific clustering, but much less pronounced than for the growing communities (Fig. 3C), as illustrated by the lower variance explained compared to the growing communities (*R*^2^ = 0.2290 and *R*^2^ = 0.4752, respectively). When each plant was analysed individually, both growth groups had similar variance explained by seasonal variations (i.e., winter, spring, summer and autumn) in all WWTPs (Figs. 3B, D).

### The dynamics of seasonal cohorts vary between growth groups but are similar between WWTPs

To explore seasonal dynamics in WWTPs, a total of 2546 species with a relative read abundance higher than 0.05% in at least one sample were analysed. 502 species were shared across all WWTPs, constituting an average cumulative read abundance of 70.0 ± 1.3% in all WWTPs. A total of 1254 species were only detected in one WWTP, constituting an average cumulative read abundance of 4.5 ± 1.0%. The rest of the species were observed in two or three WWTPs (Fig. S7). The detailed study of individual species dynamics showed that about 75% of all species had a significant seasonal component. This was independent of whether they were high-abundant (maximum relative abundance ≥ 1%), low-abundant (maximum relative abundance < 1%), or unique (i.e. species observed in only one WWTP) (Fig. S8). The seasonal strength varied among species and WWTPs. On average, 30 species showed a very strong seasonal response, 296 strong, 838 moderate, 738 weak, and 14 very weak.

**Fig. 4:**
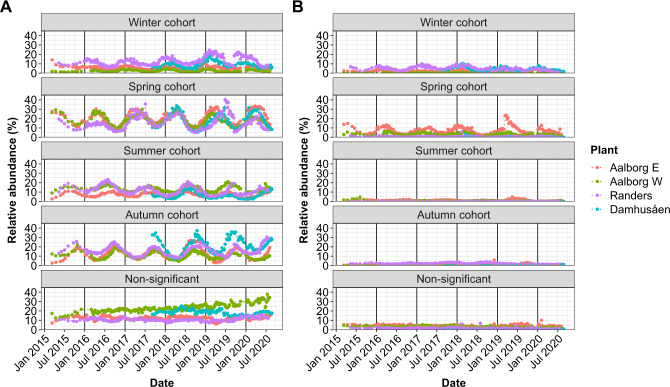
Time-series of seasonal cohorts in each WWTP. Overview of the cumulative relative read abundance of the seasonal cohorts (winter, spring, summer, autumn and non-significant) in each WWTP. **A** Growing species, **B** disappearing species. Seasonal cohorts of ambiguous and surviving bacteria can be found in Supplementary Fig. S11.

**Fig. 5:**
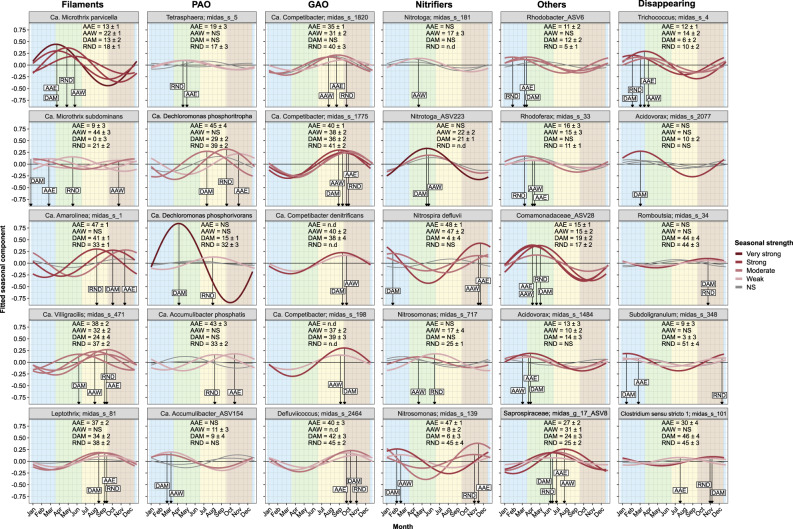
Fitted seasonal component of the top five species in the main functional guilds. Each WWTP is denoted by its initials (AAE Aalborg E, AAW Aalborg W, DAM Damhusåen, RND Randers). Colour intensity of the fitted trends represents the seasonal strength of each species in the WWTP (darker intensity represents a stronger seasonal component, grey colour represents non-significant seasonality). Background colours show the astronomical seasons for the northern hemisphere (winter, spring, summer and autumn). The text box shows the week at  maximum read abundance with the 95% confidence interval.

The seasonal strength was independent of the growth group or functional guild (Fig. S9). The classification of species into seasonal cohorts (i.e., species that peak in specific seasons) showed that the growing fraction contained a high number of species belonging to summer and autumn cohorts (Fig. S10). This is consistent with the dynamics of the estimated number of ASVs along the year. In contrast, the distribution of the cohorts in terms of relative read abundance was even between spring, summer, autumn, and non-significant cohorts (Fig. 4A). This explains why the growing fraction rarely showed a significant yearly variation, since aggregating species with different seasonality, but evenly distributed abundances can balance each other. The difference between WWTPs for the disappearing fraction (Fig. 4B) was related to the classification of the most abundant disappearing species, i.e., *Trichoccocus* midas_s_4, where the estimated peak abundance was found near the winter and spring split (Fig. 5). The time-series of seasonal cohorts of ambiguous and surviving species are shown in Supplementary Fig. S11.

The seasonal patterns of functional guilds showed a mixed response depending on the functional guild and WWTP (Figs. 5 and S12). For some functional guilds, a similar pattern was observed in all WWTPs. For example, glycogen accumulating organisms (GAO) were more prevailing in summer and autumn, while for nitrifiers and filaments, the seasonal response depended on the WWTP spanning from spring to autumn (Figs. S12 and S13). The PAOs lacked significant seasonal patterns in most of the plants, although some PAO species such as *Ca*. Dechloromonas phosphoritropha (previous MiDAS 3 placeholder name *Dechloromonas* midas_s_96) showed similar seasonal patterns in the surveyed WWTPs (Figs. 5 and S14). Therefore,  the overall seasonal pattern of guilds, or the lack thereof, depended on the actual composition and diversity of individual species within the functional guilds, and detailed exploration at species level is warranted (section “Seasonal dynamics at species level”).

### Seasonal dynamics at species level

The detailed study of seasonality at species level showed that species belonging to the same functional guild did not necessarily follow the same seasonal dynamics (Figs. 5 and S12). The same applies when aggregating species at higher taxonomic ranks (e.g., genus, family or higher), since different species in the same group showed diverse seasonal patterns. Significant seasonal patterns could be found for higher taxonomic ranks (e.g., families), but they failed to represent all species within the rank since the overall group seasonal pattern was driven by few dominating species obscuring the dynamics of the less abundant ones. Different seasonal dynamics were also found for species in the same genus. For example, the two main species of *Ca*. Microthrix, i.e., *Ca*. M. parvicella and *Ca*. M. subdominans, showed very different patterns (Fig. 5). *Ca*. M. parvicella showed a strong seasonality increasing from early to late spring, while *Ca*. M. subdominans showed a weaker seasonality with maximum peaks varying from plant to plant.

Among nitrifying bacteria, *Nitrosomonas* was the only identified ammonia-oxidising genus, and various *Nitrosomonas* species coexisted in each WWTP. However, the dominant species differed between plants and different species were transiently abundant, and those transient species did not show systematic seasonal patterns (Figs. S12 and S15). *Nitrosomonas* midas_s_717 showed a weak to non-significant seasonality, slightly peaking in spring, whereas *Nitrosomonas* midas_s_139 showed moderate to strong seasonality thriving during late autumn and winter depending on the WWTP (Fig. 5). In contrast, *Nitrosomonas* midas_s_723, that was only abundant in Damhusåen and Randers, showed similar seasonal patterns to *Nitrosomonas* midas_s_139 (Fig. S15, Supplementary file S1). *Nitrotoga* and *Nitrospira* represented the nitrite-oxidising bacteria (NOB). *Nitrotoga* midas_s_181 showed a weak to non-significant seasonality, whereas a *Nitrotoga-*related ASV (ASV223) showed stronger seasonality peaking in late spring. *N. defluvii* showed a seasonal response prevailing during late autumn to winter in the WWTPs where it coexisted with *Nitrotoga* species (Figs. 5 and S15)

These were not isolated examples since for genera with more than one species (362 out of 967), only 28% had all species classified into the same seasonal cohort, and the rest was classified into two or more seasonal cohorts (Fig. S16, Supplementary file S1). On the contrary, the GAO *Ca*. Competibacter (Figs. 5 and S12) is an example where all species in the same genus showed a very similar pattern, which was also similar across WWTPs. Damhusåen WWTP harboured the highest diversity and abundance of *Ca*. Competibacter species with estimated peaks yearly recurring between August and November, depending on the species. Additionally, species seasonality was highly reproducible among WWTPs, yet significant variations in maximum peak estimates could be found for some species (see *Ca*. Competibacter midas_s_1820 in Fig. S13). Detailed seasonal estimates for all species in each WWTP are shown in Supplementary file S1.

**Fig. 6:**
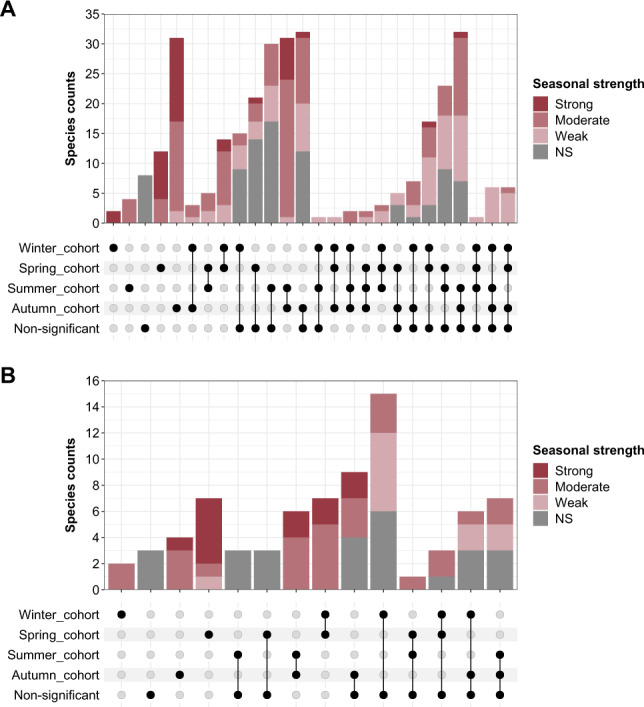
Comparison of species assigned into seasonal cohorts. Distribution of shared species between WWTPs across seasonal cohorts by growth group. **A** Growing fraction, **B** Disappearing fraction. The dotted chart represents all the possible intersections among the seasonal cohorts where the bar chart plot shows the number of species found in each intersection coloured by their seasonal strength. Colour intensity in the top bars represents the seasonal strength of the shared species in each cohort intersection, grey colour represents non-significant seasonal species. Ambiguous and surviving bacteria can be found in Supplementary Fig. S17.

The shared species among the four WWTPs (502 out of 909) were used to evaluate the repeatability of species seasonal patterns in different plants. The intersection plots (Figs. 6 and S17) show the co-occurrence of species in the seasonal cohorts among the four plants, where the dotted chart represents the intersection between seasonal cohorts and the bar chart shows the number of species found in each intersection. For both growth groups (growing fraction and disappearing fraction), most species with a very strong to moderate seasonality were assigned to the same seasonal cohort, or concomitant cohorts, in the four WWTP, corresponding to 32.5% of the shared growing species and 34.2% of the shared disappearing species (Fig. 6). This phenomenon summarises the visualisation dynamics in Fig. 5, where species with a strong seasonal component, such as *Ca*. M. parvicella, *Ca*. Amarolinea midas_s_1, *Ca*. Competibacter midas_s_1775, *Comamonadaceae* ASV28 or *Trichococcus* midas_s_4, among others, tended to peak during the same season or concomitant seasons in all WWTPs. The intersections where species were distributed within 2 or 3 cohorts plus the non-significant cohort, capture the variability observed for some species (31.2% of the shared growing species and 17.1% of the shared disappearing species). These species tended to show a weak to moderate seasonal component and the estimated relative read abundance peak spanned over several months depending on the WWTP (e.g., *Ca*. D. phosphoritropha, *N. defluvii*, or *Clostridum* sensu stricto 1 midas_s_101, Fig. 5). For the WWTPs studied, no species were classified into opposite cohorts. Finally, some species were exclusively found in the non-significant cohort (eight growing species and three disappearing species) suggesting that their abundance in the WWTPs was not affected by any seasonally periodic component (Fig. 6, Supplementary file S1).

## Discussion

Our longitudinal survey of four full-scale WWTP with nutrient removal during 3–5 consecutive years with a new approach, including time-series decomposition and ecosystem-specific species-level classification, showed significant seasonal dynamics for about 75% of the identified species. The seasonal dynamic was cyclic (i.e., repeating each year) and comparable across WWTPs with similar process configuration. Moreover, our approach allowed us to evaluate if distinct seasonal patterns could be observed for bacterial species depending on their functional guild, level of taxonomic aggregation, and net growth rate in the AS bioreactor.

Interestingly, the growing and disappearing growth groups exhibited different alpha- and beta-diversity patterns. In particular, the beta-diversity of disappearing microbial communities resembled the beta-diversity commonly observed in influent wastewater communities [4, 43]. This can be explained by the fact that disappearing species in AS are only present as a consequence of mass immigration of bacteria with the influent wastewater [4]. The beta-diversity of growing and disappearing groups in each WWTP showed distinct clustering patterns (PCA, Fig. 3). This indicated that each WWTP had its own signature microbial community, where differences were stronger for the growing bacteria than for the disappearing species. However, the top abundant species in each growth group were the same across all four WWTPs and they were similar to other Danish plants with nutrient removal [18]. This suggests that the clustering was determined by differences in their relative read abundances as well as differences in low-abundant species. Both growth groups showed a seasonal pattern, but they showed a different seasonal response. The growing group was characterised by a higher number of low-abundant species peaking during the summer and autumn cohorts. However, the cumulative relative read abundance explained by species within each seasonal cohort showed an even distribution. In contrast, the species in the disappearing group were more prevalent during winter/spring, indicating the influence of different seasonal drivers affecting the species in the two growth groups. Since the disappearing group consists of only immigrating bacteria that do not thrive in the process tanks, their abundance must be controlled by upstream factors in the sewer system and by factors affecting their degradation rate in the AS.

Growing bacteria are assumed to be active in the AS system and perform process-critical functions [21, 44], so a common approach is to group microorganisms according to their potential functionality (e.g., nitrifiers or PAO) or similar morphology (e.g., filamentous organisms) to evaluate WWTP performance. However, these guilds may encompass species from different phylogenetic groups and have diverse metabolic potentials. Some guilds showed an overall seasonal pattern, but the results should be extrapolated with great caution since the dynamics depended on the actual composition, diversity, and abundance of species within the defined guild. For example, in Damhusåen WWTP, the two main filamentous species *Ca*. M. parvicella (spring cohort) and *Ca*. Amarolinea midas_s_1 (autumn cohort) showed a similar mean relative read abundance over the years (~5.7% and 4.0%, respectively), hence the filamentous guild did not show any overall seasonality. Distinguishing the individual taxa dynamics beyond the overall guild has great practical implications since these two filamentous bacteria are the main responsible taxa for poor sludge settling and filamentous bulking, and the different physiologies may require distinct control measures [20, 45]. However, filamentous bacteria share a morphological trait rather than a metabolic trait, therefore, the different species might not occupy the same niche and compete for the same resources. Therefore, the identity of species in these groups may vary depending on the geographical location of the WWTP and environmental conditions [1, 46].

Nitrifiers form a well-described and defined guild with few genera abundant in WWTPs worldwide, i.e., *Nitrosomonas*, *Nitrotoga* and *Nitrospira* [1]. In our study, all WWTPs reported a good nitrogen removal performance [24, 25, Fig. S18, Supplementary files S2 and S3] and showed a comparable relative read abundance of nitrifiers (0.8–2.0%) with some fluctuations during the years (Fig. S15). Different species of *Nitrosomonas* coexisted in the WWTPs and some showed seasonal patterns. However, both the seasonal response and the dominant *Nitrosomonas* species varied between plants, making it difficult to elucidate the drivers for each species. The dominant NOB was also WWTP dependent. In the Randers plant only *N. defluvii* was above the quantification limit (but *Nitrotoga* could be detected with a read abundances below 0.01%), while in the other WWTPs two *Nitrotoga* species coexisted with seasonal increases in *N. defluvii*. Intriguingly, within the plants where different NOB coexisted, *N. defluvii* showed a similar seasonality increasing from late autumn to late winter in all WWTPs concomitant with lower read abundances of *Nitrotoga* species. Based on genomic studies, *Nitrotoga* and *Nitrospira* have diverse metabolic potential as well as different membrane-bound orientations of the nitrite oxidoreductase enzyme, indicating different affinities in nitrite uptake [47, 48], which suggests the possibility of coexistence. The long-term dynamic pattern observed between *Nitrotoga* and *Nitrospira* species in different years and WWTPs, suggests that for a given WWTP nitrification capacity, some degree of competition occurs between these NOB genera. However, as discussed below, immigration may partly be responsible for these dynamics, so further studies are necessary to determine the factors influencing species seasonality and the practical implications for the WWTP operation.

Many species showed similar seasonal patterns across the four WWTPs suggesting the influence of some overarching factors (Figs. 5 and 6). Variations in process parameters could explain some of the species’ observed variance (Supplementary Note 1, Figs. S19 and S20). However, most of the measured process parameters such as influent chemical oxygen demand, ammonia or phosphate, among others, fluctuated randomly during the years suggesting a minor impact on the observed seasonal dynamics (Supplementary Note 1, Supplementary files S2 and S3). Temperature was the only measured parameter that showed a recurrent seasonal pattern across the WWTPs (Supplementary file S4, Supplementary Note 1). Process tank temperature has been suggested as the most important factor to explain seasonal dynamics in WWTPs [6, 8, 9, 11–13]. Temperature affects growth and decay rates, degradation rates and biochemical transformations, or liquid-gas solubility among others, hence having a profound impact on species ecophysiology. Some ecophysiological characteristics can be found for pure cultures, but they are rarely described in situ, making it very difficult to associate the impact of temperature over individual species. Nevertheless, temperature alone cannot explain all the different seasonal dynamics observed, nor the variability for some species between WWTPs. For example, the maximum relative abundance peak estimates could vary about 2 months between WWTPs, even for strongly seasonal species (e.g., *Ca*. M. parvicella, spring cohort), while for weakly seasonal species even greater variability could be observed (e.g., *Ca*. M. subdominans). Substrate availability and composition is another important factor that can affect seasonal dynamics [11, 49, 50]. For example, in combination with temperature, a seasonal influent lipid loading was found to increase the abundance of a foam-forming microorganism related to *Gordonia*, causing seasonal bulking [49]. Other factors, such as SRT, are typically considered in WWTP operation since SRT is inversely proportional to net microbial growth rates [51], but it is unclear if typical SRT fluctuations in WWTP can apply enough selective pressure to influence strong seasonal microbial dynamics. Indeed, full-scale experiments have shown minor differences in bacterial classes between an SRT of 12 and 30 days [52], which is a common operational range for many municipal AS bioreactors in temperate climates.

Immigrating bacteria with influent wastewater could also affect the observed seasonal dynamics, although their contribution to seasonality in AS has not yet been studied in detail. Bacteria from the source communities (e.g., sewer systems) are continuously added to the AS, and recent longitudinal studies have shown some seasonal patterns in sewer and AS influent microbial communities [12, 53, 54]. The influence of mass immigration is clear for the disappearing group, which dies in the AS tank and it is only present due to the wastewater inflow. A good example is *Trichococcus*. *Trichococcus* has been reported to be more abundant in the sewer systems in colder climates and during colder months [53, 54], which may explain our observations of the strong dynamics of *Trichococcus* showing a maximum seasonal peak late winter and early spring. This supports the findings that abundant species in AS are influenced by their abundance in the influent wastewater. The influence of mass immigration can be extended to the growing fraction, where most species are expected to perform process-critical functions in the AS. The AS growing species are generally present in low abundance in the influent wastewater [4], and their abundance may also be seasonal in the influent wastewater and thereby determine, at least partly, the AS seasonal dynamic. However, to date, this is undescribed. Proving a mechanistic causation of specific drivers for each species is challenging. However, the combination of targeted experiments of the microbial immigration, the chemistry of influent wastewater, the application of deterministic and stochastic models, or deep learning may in the near future allow a better understanding of the factors that drive the species dynamics.

The observed recurrent long-term seasonal dynamics of many species has several practical implications for WWTP understanding, operation and performance. Importantly, we have shown that seasonal dynamics are species-specific and some variations exist among WWTPs without clear correlations to the process parameters. Therefore, WWTPs should analyse their community pattern at species level by standardised taxonomy (e.g., MiDAS 4) for at least 2 years to establish a “normal” baseline for the plant, concomitantly with a detailed surveillance of the process and operational parameters and influent wastewater. This will allow improving full-scale experiments design, results interpretation and comparability among studies. Currently, this is, in general, far beyond normal practice. Alternatively, when carrying out experiments in full-scale WWTPs, replicate bioreactors (i.e., independent parallel AS lines) could be used as seasonal control, although this option may not be feasible for most WWTPs. Looking beyond, where on-site or “online” sequencing is implemented with the control system of full-scale AS bioreactors, seasonal dynamics will need to be considered to develop robust and effective process control loops.

## Supplementary information

Supplementary material

Dataset1

Dataset2

Dataset3

Dataset4

## Data Availability

Amplicon sequencing data is deposited in NCBI project PRJNA757616.
