# Supplementary material for: Microbial communities across activated sludge plants show recurring species-level seasonal patterns
Source: ISME Commun. 2022 Feb 18;2:18. doi: 10.1038/s43705-022-00098-4 (PMC9723569; doi:10.1038/s43705-022-00098-4)
Supplement: Supplementary file 1 — Supplementary material [file 43705_2022_98_MOESM1_ESM.pdf]

Supplementary material

Microbial communities across activated sludge plants show recurring species-level seasonal patterns

Miriam Peces[1], Giulia Dottorini[1], Marta Nierychlo[1], Kasper Skytte Andersen[1], Morten Kam Dahl Dueholm[1], Per Halkjær Nielsen[1,*]

[1] Department of Chemistry and Bioscience, Section of Biotechnology, Center for Microbial Communities, Aalborg University, Aalborg East, 9220, Denmark

* Corresponding author: Per Halkjær Nielsen, Center for Microbial Communities, Department of Chemistry and Bioscience, Aalborg University, Fredrik Bajers Vej 7H, 9220 Aalborg, Denmark. Phone: +45 9940 8503; E-mail: phn@bio.aau.dk

Table of contents

Table S1. Main process characteristics of the surveyed WWTPs 2
Figure S1. Distribution of total reads per sample 2
Figure S2. Approach to determine species seasonality in WWTPs. 3
Figure S3. Yearly process tank temperature in the four Danish WWTPs 4
Figure S4. Distribution of species seasonal strength 4
Figure S5. Overview of top abundant species in the ambiguous and surviving growth groups 5
Figure S6. Yearly median alpha-diversity estimates in the four WWTPs 5
Figure S7. Shared species among the four WWTPs 6
Figure S8. Species distribution into seasonal cohorts 7
Figure S9. Seasonal strength distribution of species grouped by seasonal cohorts 8
Figure S10. Distribution of seasonal cohorts in the four WWTP 9
Figure S11. Time-series of seasonal cohorts in each WWTP 10
Figure S12. Overview of time-series and seasonal distribution of functional guilds 11
Figure S13. Detailed study of the dynamics of the GAO guild 12
Figure S14. Detailed study of the dynamics of the PAO guild 12
Figure S15. Detailed study of the dynamics of the nitrifier guild: AOB and NOB species 13
Figure S16. Classification of species within the same genus into seasonal cohorts 14
Figure S17. Comparison of species assigned into seasonal cohorts 15
Note 1: Species response to operational and environmental variables 16
N.1.1 Main monitored parameters 16
Figure S18. Visualisation of main monitoring parameters in each WWTP 17
N.1.2 Variance partitioning at species-level and seasonality of operational variables 18
Figure S19. Explained variance of the monitoring parameters for all growing species 19
Figure S20. Explained variance of the monitoring parameters for individual species 21
References 21

Table S1. Main process characteristics of the surveyed WWTPs

Plant	Design size (PE)	Type	Anaerobic Configuration	Anoxic/ Aerobic Configuration	SRT (days)	Industrial load (% COD)	Industry	Sampling Period	Number of samples
Aalborg E	150 000	N-removal and EBPR	SSH	Alternating	(10 – 25)	25%	Abbatoir / Chemistry	2015 – 2020	223
Aalborg W	330 000	N-removal and EBPR	SSH	Alternating	(10 – 25)	25%	Dairy	2015 – 2020	251
Damhusåen	350 000	N-removal and EBPR	MAT	Alternating	(9 – 32)	15 – 20%	Various	2017 – 2020	146
Randers	130 000	N-removal and EBPR	SSH	Recirculation	(10 – 35)	5%	Dairy / Food	2015 – 2020	243

PE: Person equivalent; SRT: Solid retention time; COD: Chemical oxygen demand; SSH: Return sludge side-stream hydrolysis; MAT: Main-stream anaerobic tank

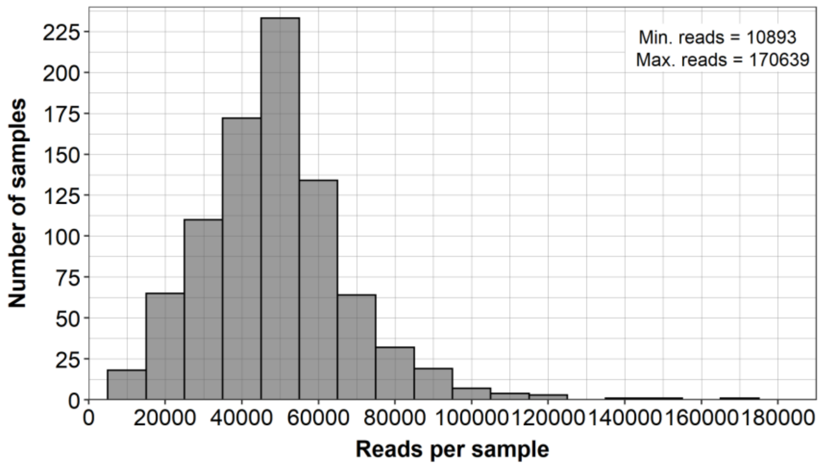

Figure S1. Distribution of total reads per sample

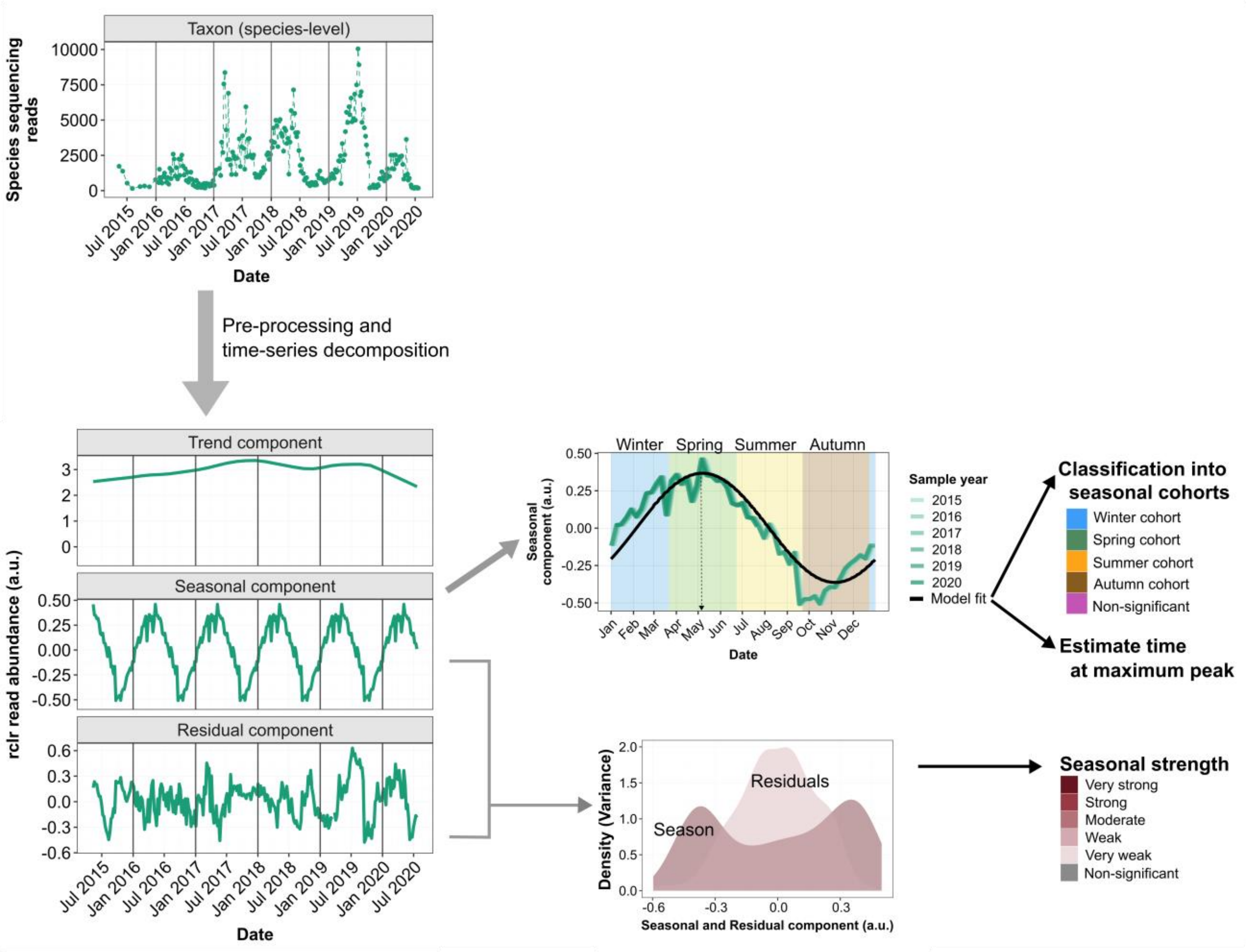

Figure S2. Approach to determine species seasonality in WWTPs. Overview of the approach used in this study as detailed in section 2.3.2 of the main manuscript.

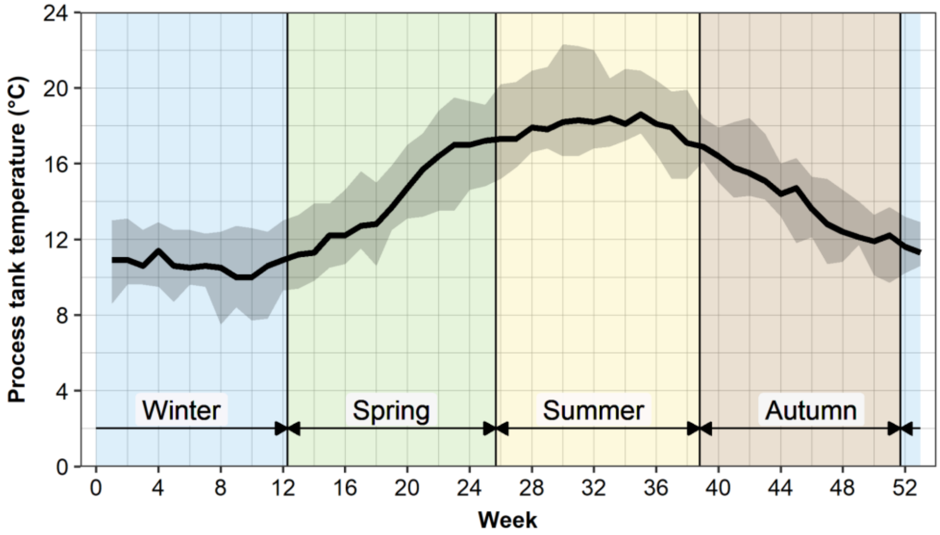

Figure S3. Yearly process tank temperature in the four Danish WWTPs. Process tank temperature profile overlapped with the definition of astronomical seasons in the northern hemisphere used to define the seasonal cohorts.

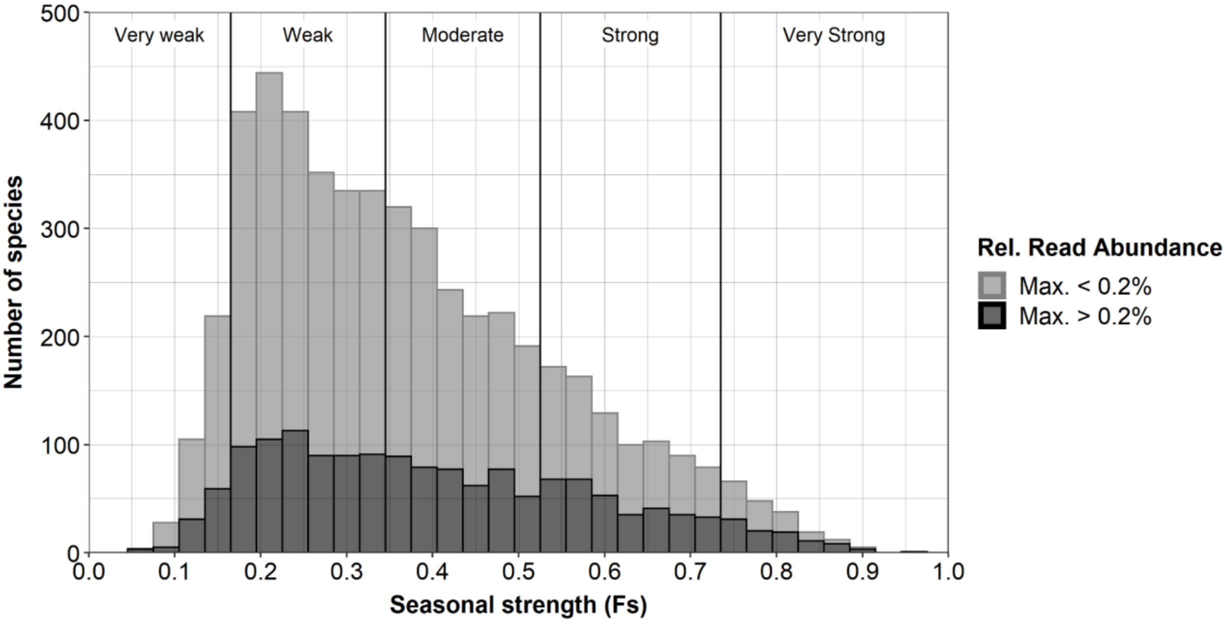

Figure S4. Distribution of species seasonal strength. Distribution of seasonal strength (Fs) in all species with a significant seasonal component ($p < 0.01$) to define the cut-offs for the seasonal strength definition, from very strong to very weak.

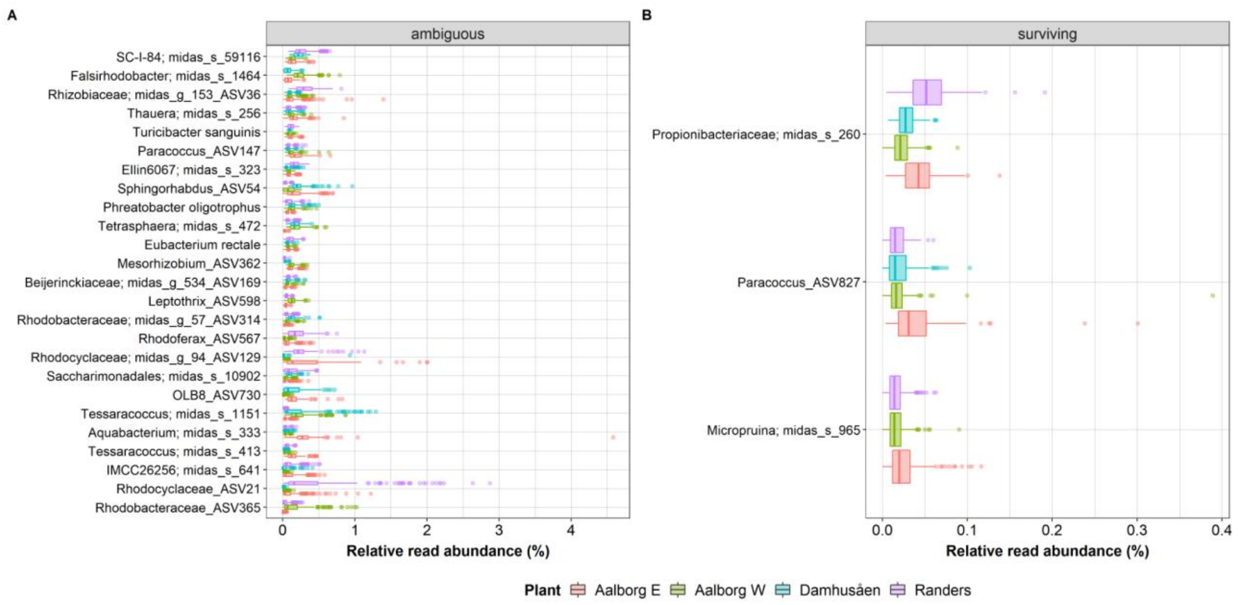

Figure S5. Overview of top abundant species in the ambiguous and surviving growth groups. Relative read abundance of the top 25 most abundant bacterial species in the four WWTPs. (A) Species in the ambiguous group, (B) species in the surviving group.

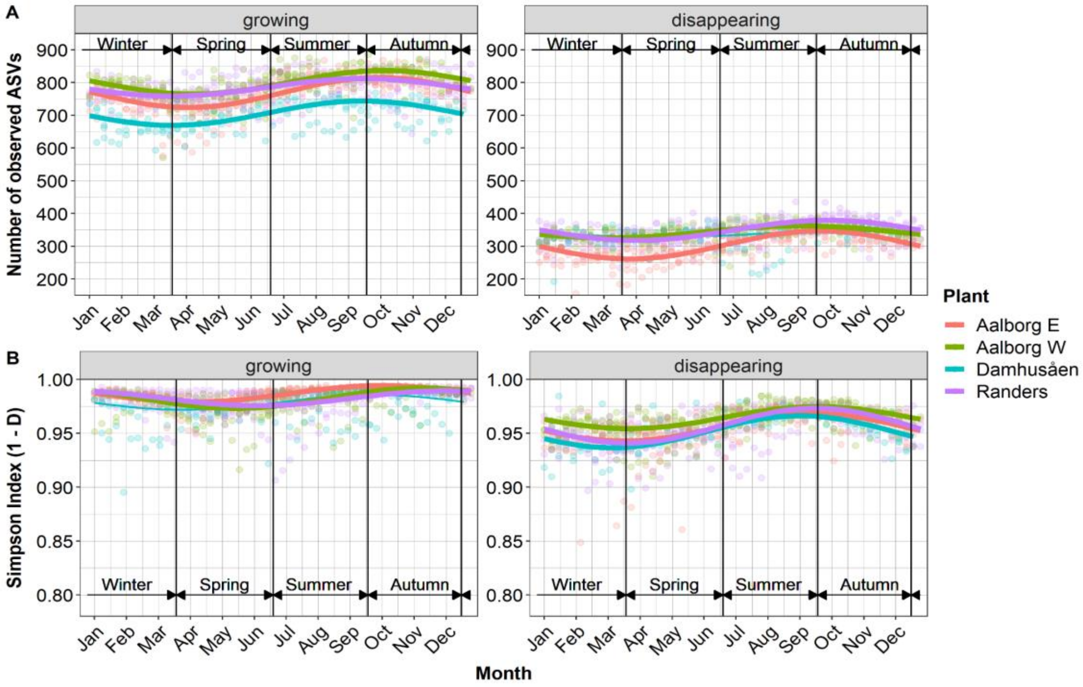

Figure S6. Yearly median alpha-diversity estimates in the four WWTPs. Alpha-diversity estimates were calculated using ASV-level taxa separated by growth group. (A) Number of observed ASVs in growing and disappearing fractions. (B) Simpson index in growing and disappearing fractions.

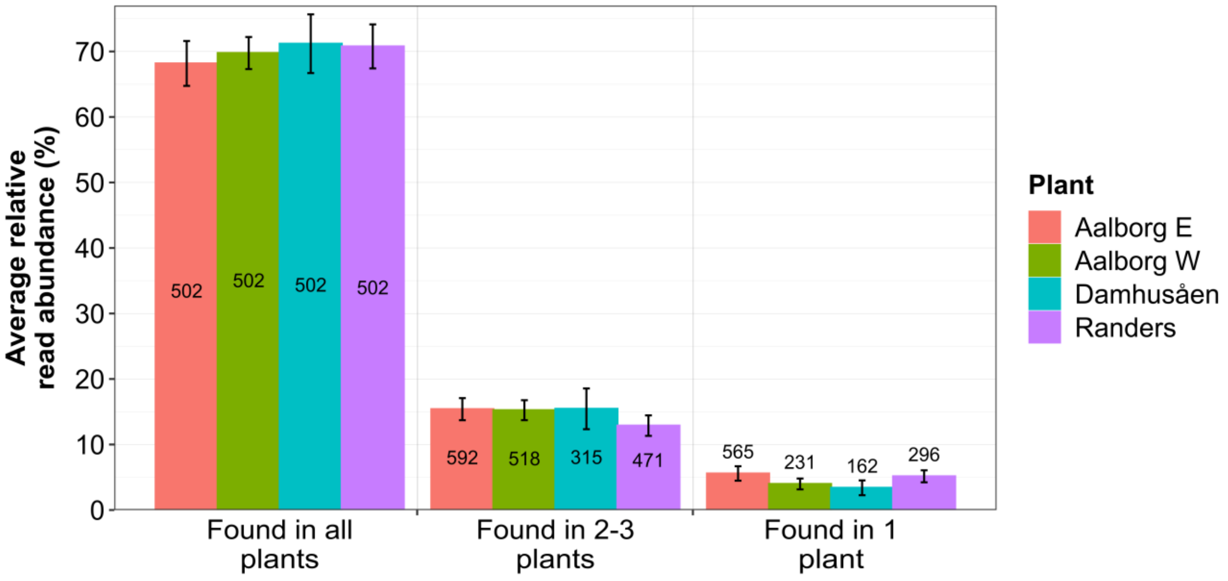

Figure S7. Shared species among the four WWTPs. Species distribution in WWTPs depending if the species were found in all WWTPs, found between 2 or 3 plants, and unique. Bars represent the average read abundance explained by those species in each WWTP. Numbers show the number of different species in each group and WWTP.

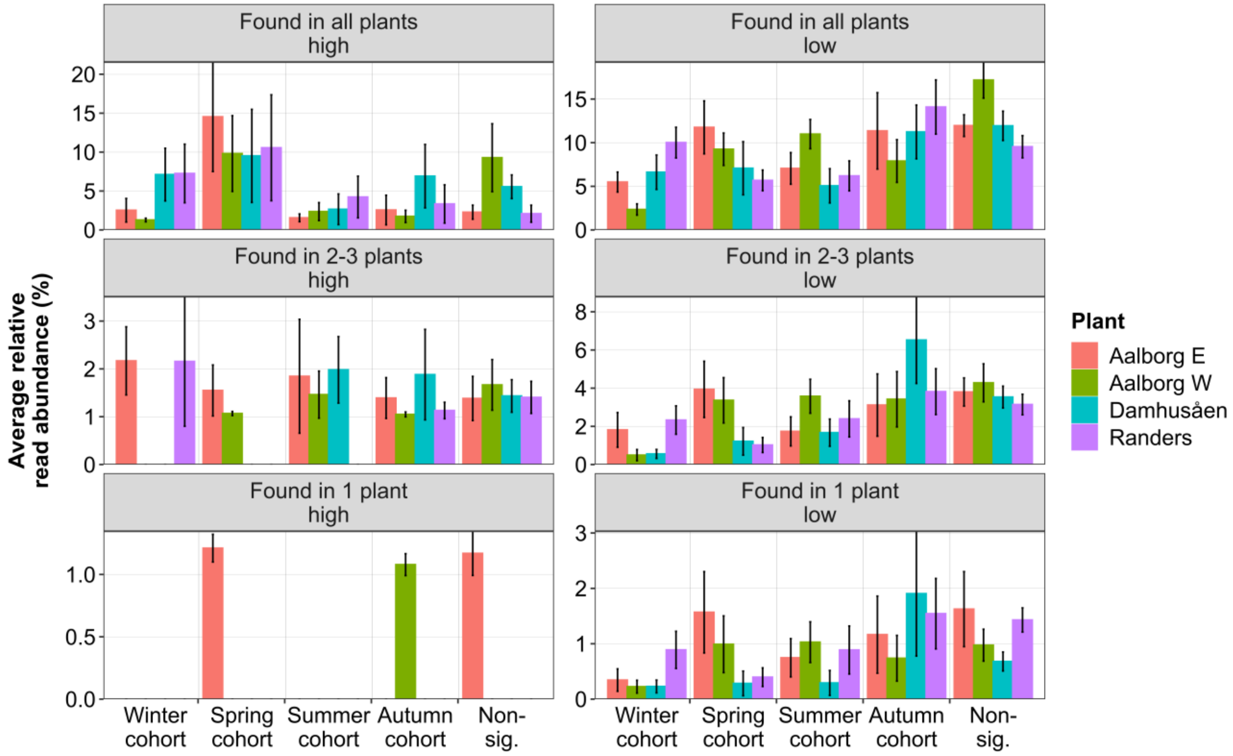

Figure S8. Species distribution into seasonal cohorts. Species distribution into seasonal cohorts for each WWTPs depending if the species were found in all WWTPs, found between 2 or 3 plants, or uniquely separated by their relative read abundance (high: >1% at least in one sample; low: < 1% relative read abundance).

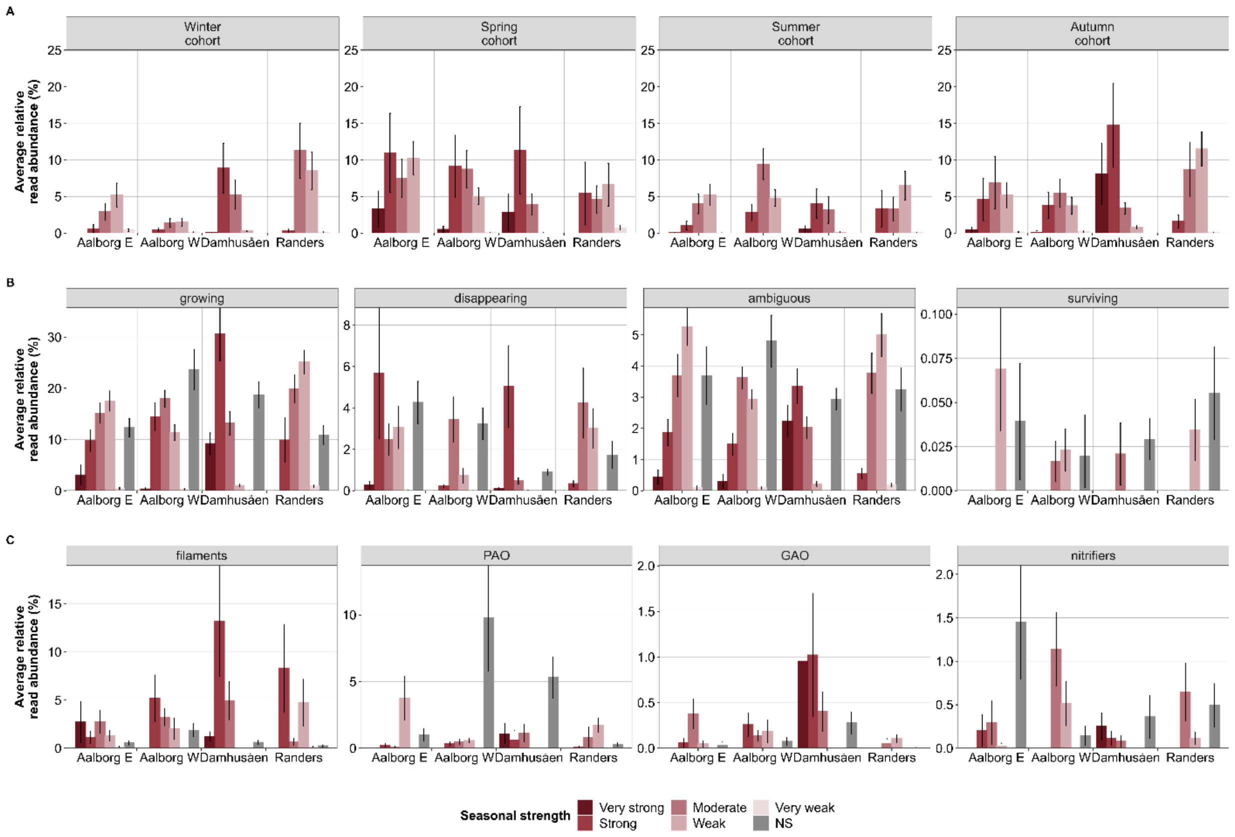

Figure S9. Seasonal strength distribution of species grouped by seasonal cohorts (A), growth groups (B), and functional guilds (C). Species seasonal strength depending on their assigned seasonal cohort, growth group or functional guild in each WWTP. Bars represent the cumulative relative abundance of the species in the particular group. Colour intensity corresponds to the seasonal strength. Grey bars summarise the non-significant seasonal species

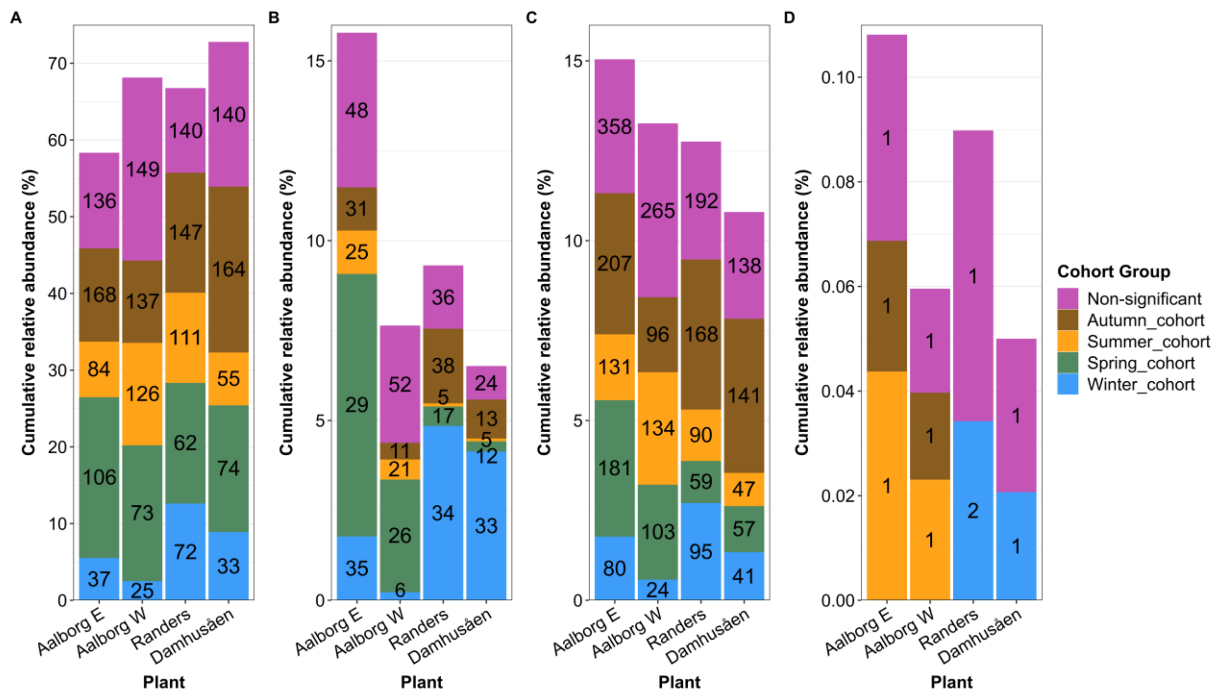

Figure S10. Distribution of seasonal cohorts in the four WWTP. Overview of the cumulative read abundance of each seasonal cohort. (A) Growing species, (B) disappearing species, (C) ambiguous species, (D) surviving species. Numbers in the cumulative relative abundance plot show the average number of different species in each seasonal cohort and WWTP.

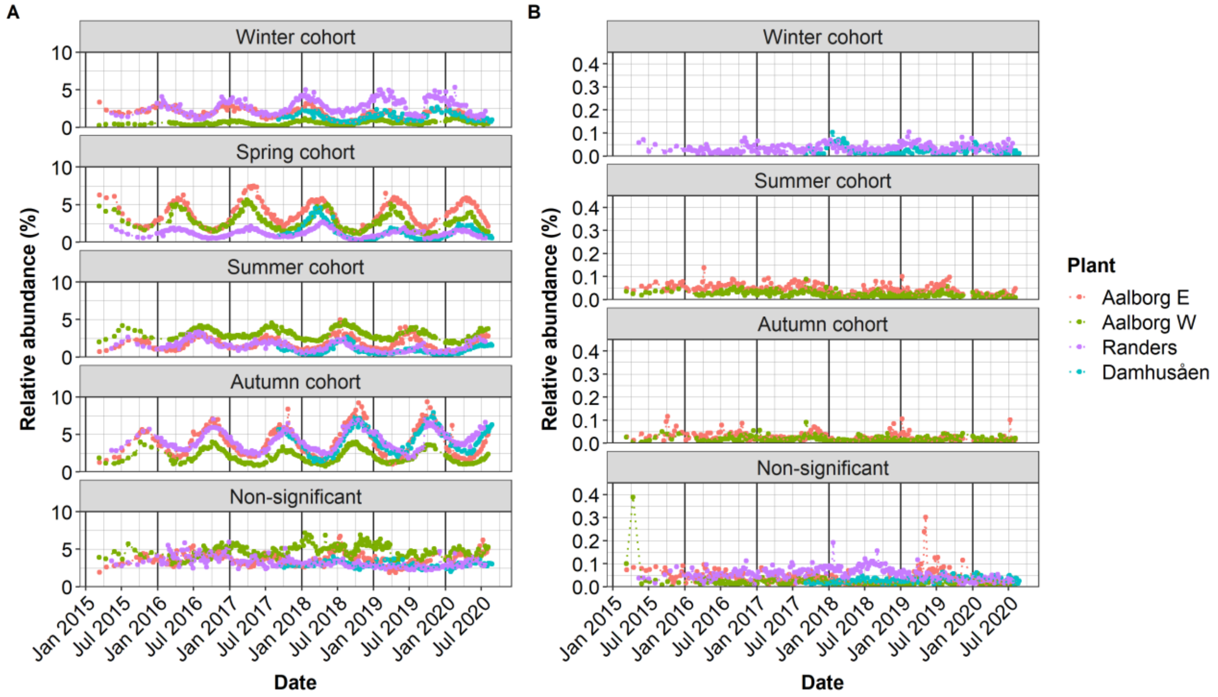

Figure S11. Time-series of seasonal cohorts in each WWTP. Overview of the cumulative relative read abundance of the seasonal cohorts (winter, spring, summer, autumn, and non-significant) in each WWTP. (A) Ambiguous species, (B) surviving species. None of surviving species were classified into the spring cohort.

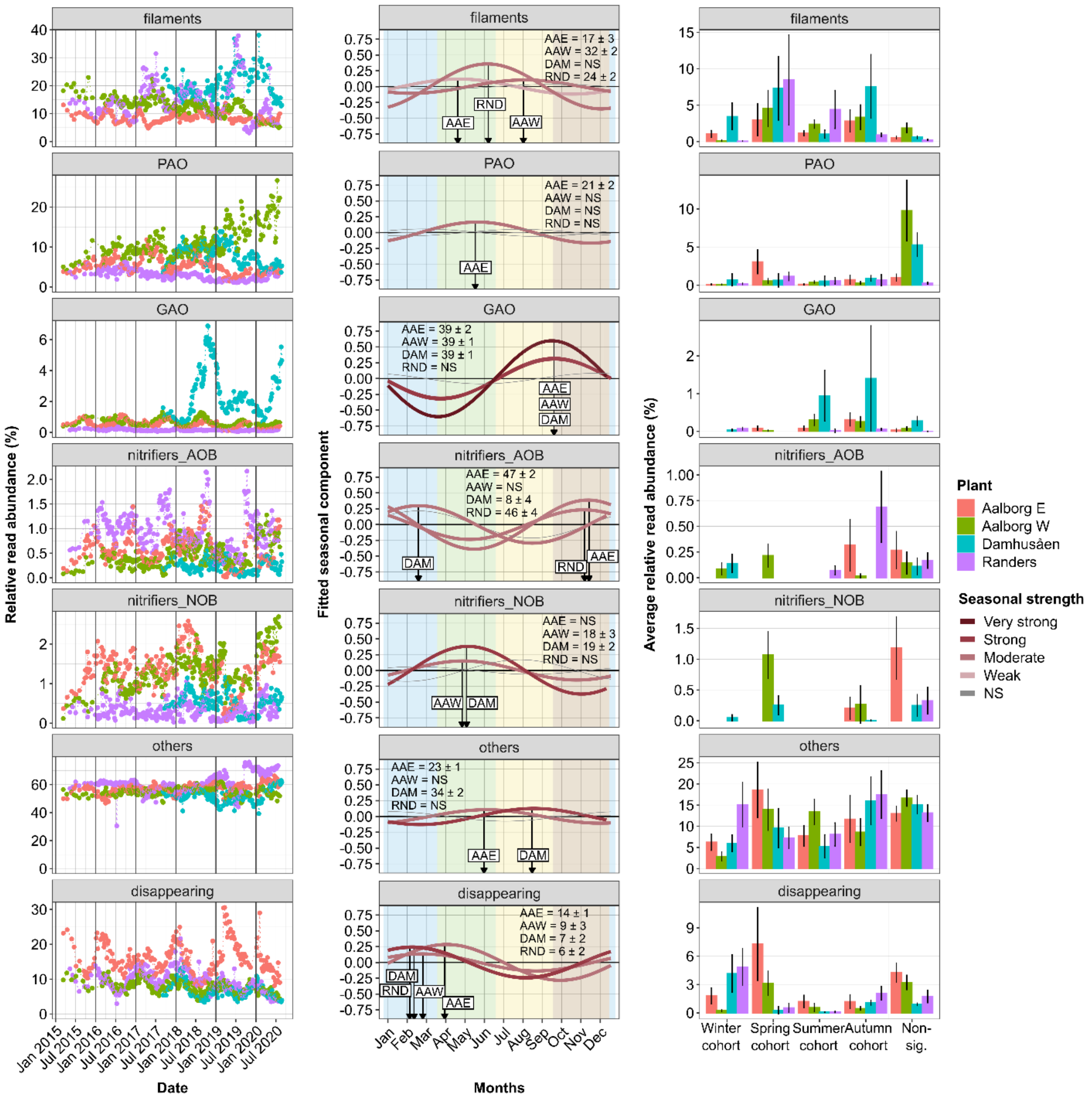

Figure S12. Overview of time-series and seasonal distribution of functional guilds. Left vertical panel: Time-series of the main functional guilds and morphological groups. Middle vertical panel: Estimation of the maximum peak time of the seasonal component. Right vertical panel: Distribution of species into the seasonal cohorts. Each WWTP is represented by its own colour code and initials (AAE = Aalborg E (red), AAW = Aalborg W (green), DAM = Damhusåen (blue), RND = Randers (purple)). Maroon colour intensity corresponds to the seasonal strength. Grey lines indicate non-significant seasonal guilds.

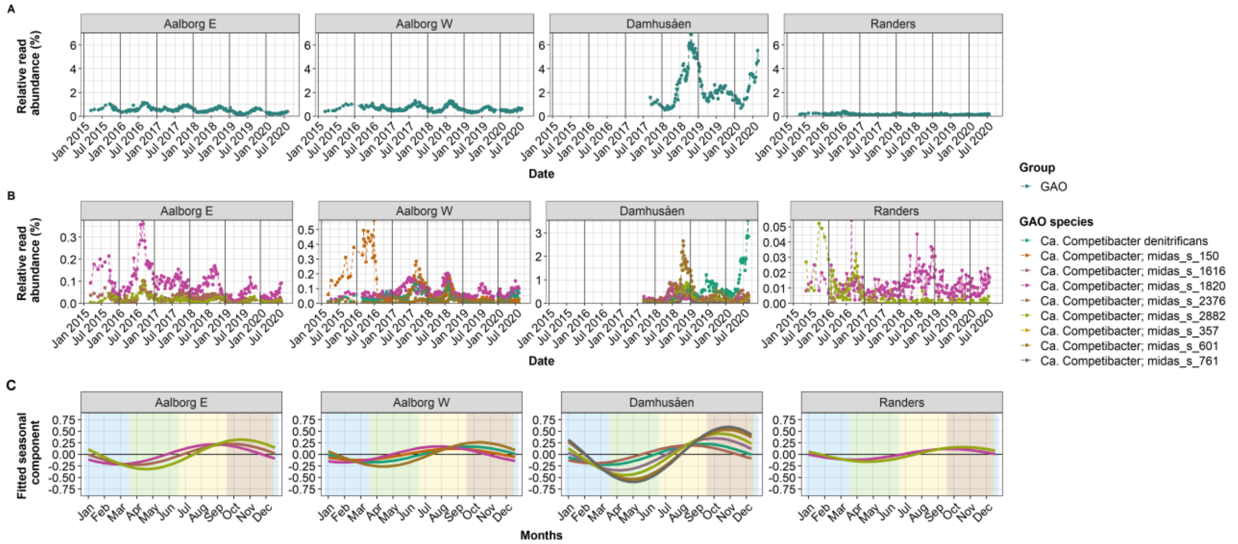

Figure S13. Detailed study of the dynamics of the GAO guild. (A) Time-series of the GAO guild. (B) Time-series of the main GAO species. (C) Seasonal dynamics of the main GAO species, thin line indicates that the seasonal component was not significant (p>0.01).

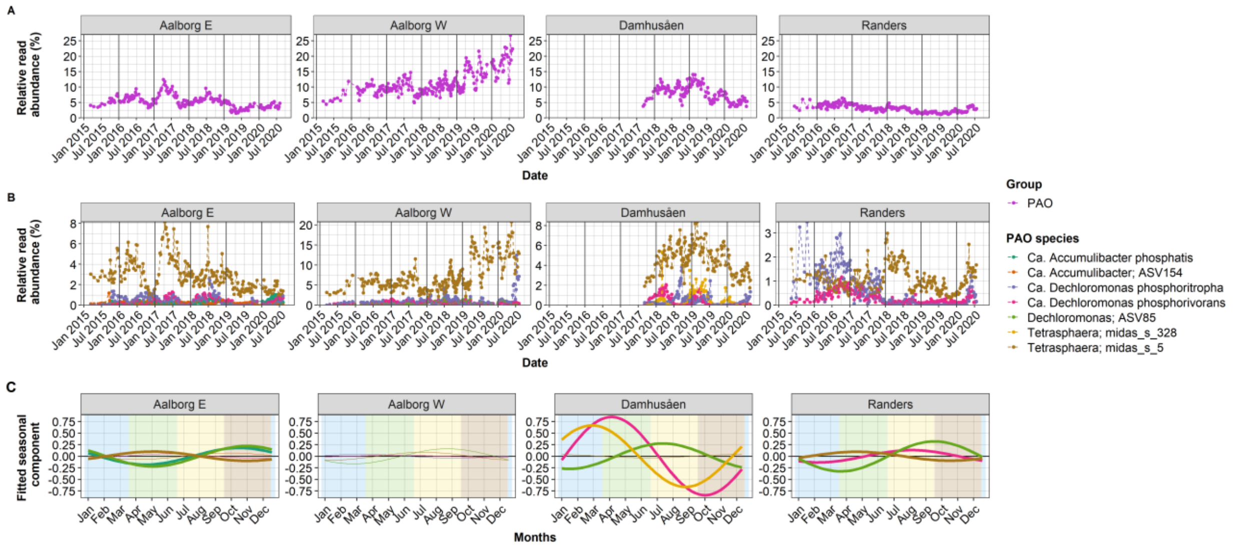

Figure S14. Detailed study of the dynamics of the PAO guild in each WWTP. Only PAO species with a relative read abundance > 1% at least in one sample are shown. (A) Time-series of the PAO guild. (B) Time-series of the main PAO species. (C) Seasonal dynamics of the main PAO species, thin line indicates that the seasonal component was not significant (p>0.01).

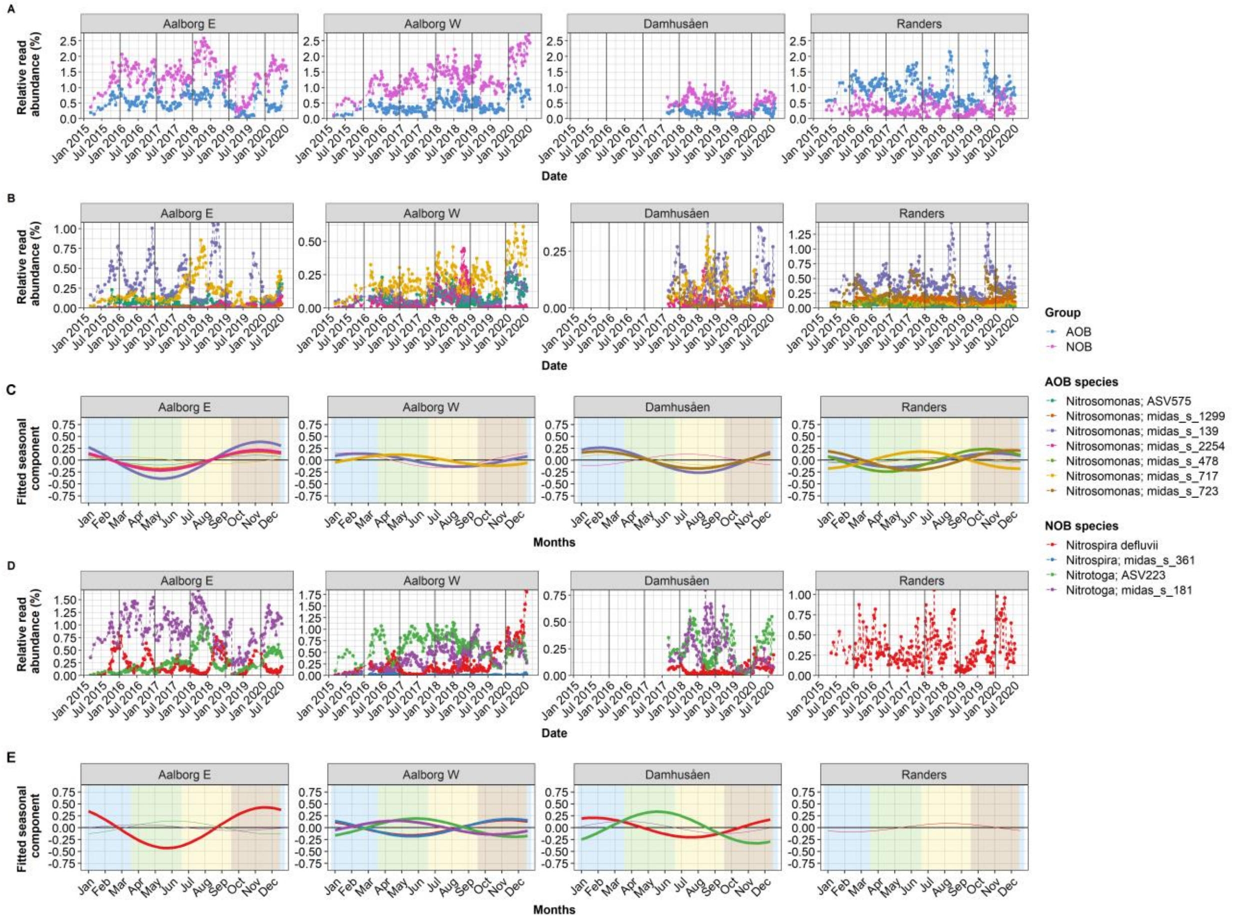

Figure S15. Detailed study of the dynamics of the nitrifier guild: AOB and NOB species in each WWTP. (A) Time-series of the AOB and NOB guilds. (B) Time-series of AOB species. (C) Seasonal dynamics of AOB species, thin line indicates that the seasonal component was not significant (p>0.01). (D) Time-series of NOB species. (E) Seasonal dynamics of NOB species, thin line indicates that the seasonal component was not significant (p>0.01).

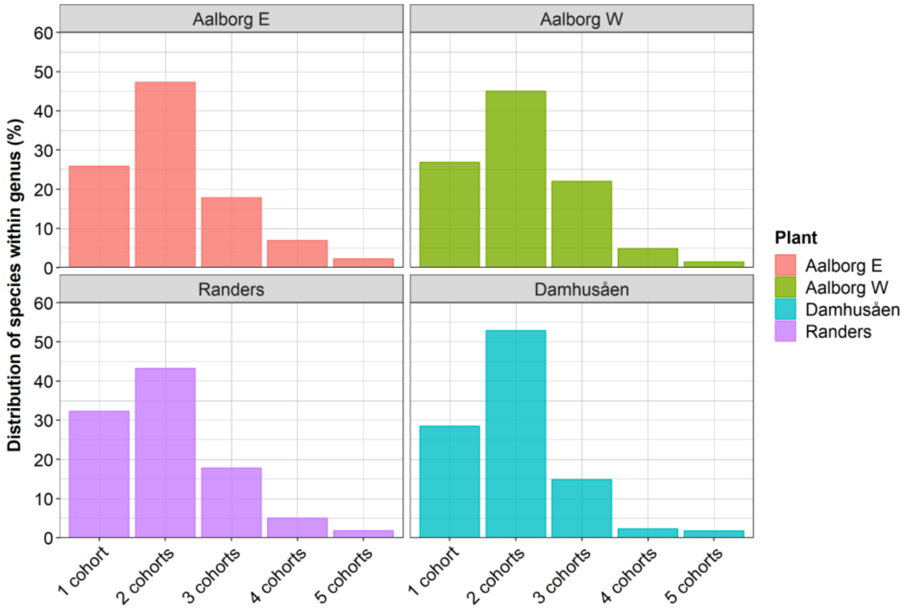

Figure S16. Classification of species within the same genus into seasonal cohorts. Distribution and classification of species into seasonal cohorts within the same genus (for genera containing more than one species) in the four WWTPs.

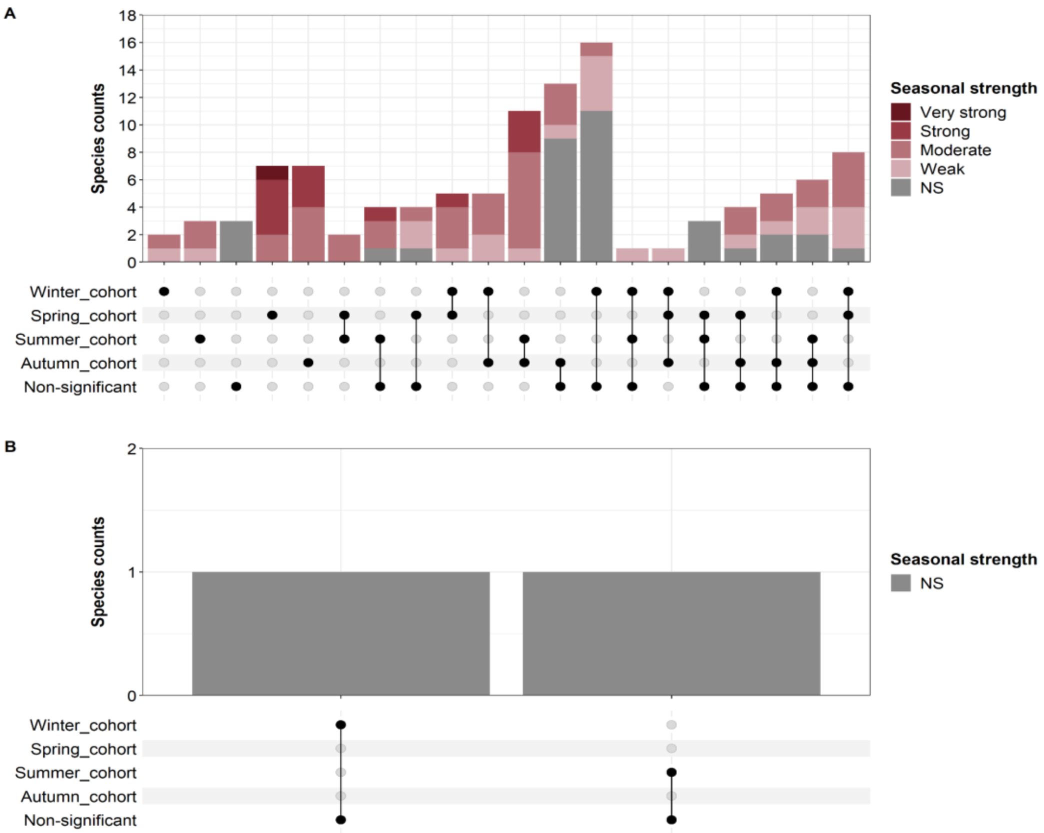

Figure S17. Comparison of species assigned into seasonal cohorts. Distribution of shared species between WWTPs across seasonal cohorts by growth group. (A) Ambiguous fraction, (B) surviving fraction. Colour intensity in the top bars represents the seasonal strength of the shared species in each cohort intersection, grey colour represents non-significant seasonal species.

Note 1: Species response to operational and environmental variables

N.1.1 Main monitored parameters

Figure S18 visualises some of the parameters in Aalborg West (**Figure S18A**) and Damhusåen (**Figure S18B**). We do not have access to similar data for Randers and Aalborg E. Aalborg West and Damhusåen show a normal process performance throughout the years. Not all the WWTP measure the same parameters, with the same frequency or the same methods, which complicates cross-WWTPs comparisons. Time-series decomposition and simple harmonic fitting were used to analyse the seasonal component of the monitored parameters. The methodology used was the same as for the species evaluation (see section 2.3.2 in the main manuscript) except the measured parameters were standardised to mean of zero and variance of 1 prior to time-series decomposition.

The results of the analysis show that most of the monitored operational parameters fluctuate randomly during the year (see **Supplementary file S4** for detailed analysis). Briefly, only temperature (both WWTPs), effluent nitrate concentration (both WWTPs), hydraulic retention time (HRT, Aalborg West), sludge volumetric index (SVI, Damhusåen), suspended solids in the tank (SS, Damhusåen), SS in the effluent (Damhusåen) dissolved oxygen (DO, Damhusåen) and influent insoluble nitrogen (Damhusåen). Wastewater temperature and effluent nitrate concentration were the only parameters that showed a similar pattern in both plants. Temperature can affect microbial growth rates, but also shift the biochemical equilibria and kinetic rates. For example, lower denitrification activity is well-known to vary depending on the organic load and the temperature in the anoxic reactor (Metcalf & Eddy et al., 2014). Higher nitrate concentration is usually observed when wastewater temperature is lower (winter in the northern hemisphere) due to lower denitrification kinetic rates (Dawson and Murphy 1972, Metcalf & Eddy et al., 2014). Moreover, lower temperatures increase oxygen solubility which makes it more difficult achieving anaerobic conditions in the anoxic tank, inhibiting denitrification (Oh and Silverstein, 1999). Other reasons for lower denitrification rates might be low organic concentration in the influent wastewater, although no systematic changes were observed for Aalborg West and Damhusåen. However, given the high diversity of potential denitrifying species, it is unclear if the lower denitrification activities are related to seasonal changes in microbial species or a kinetic response due to environmental factors where denitrification has a lower activity than nitrification.

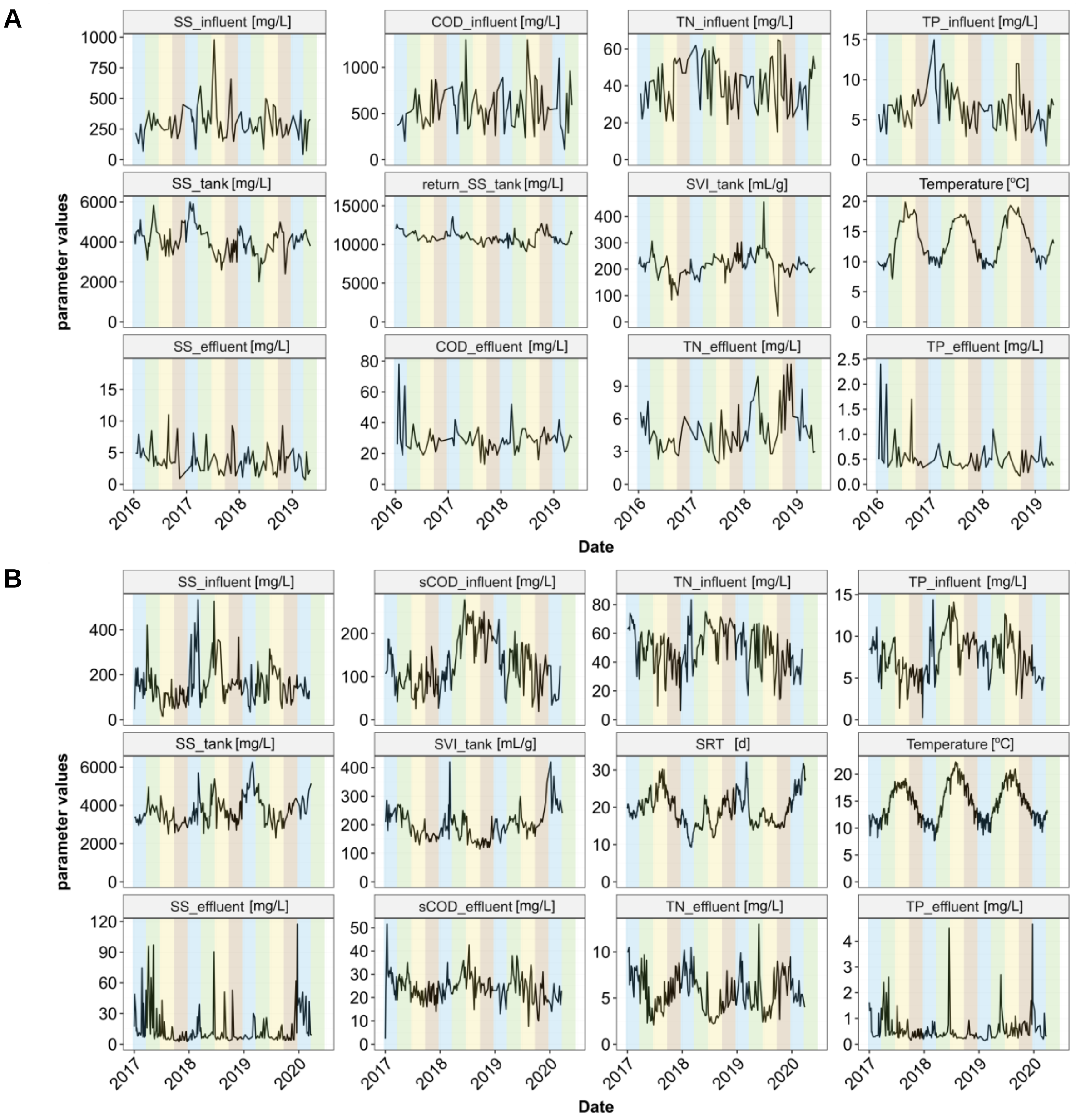

Figure S18. Visualisation of main monitoring parameters in each WWTP. (A) Aalborg West. (B) Damhusåen. Shaded coloured panels correspond to the season during the year. The complete datasets for the monitored parameters can be found in **supplementary files S2** and **S3**.

N.1.2 Variance partitioning at species-level and seasonality of operational variables

Variance partitioning analysis was used to estimate the contribution of each of the monitored parameters to the species-observed variability. Variance partitioning analysis was carried in R using the package variancePartition (Hoffman and Schadt, 2016). In case of collinear monitoring parameters, only the most commonly used has been kept. For example, chemical oxygen demand (COD), biological oxygen demand (BOD) and total organic carbon (TOC) are proxies for organic matter, and in general, are linearly correlated. For this study, we chose COD since it was measured in both plants. To remove the collinearity from total nitrogen (TN) and ammonia, and total phosphorus (TP) and orthophosphate; ammonia was subtracted from TN and orthophosphates from TP. The resulting fractions named insoluble nitrogen and insoluble phosphorus were retained together with ammonia and orthophosphate. **Figure S19** summarises the explained variance for all growing species in Aalborg West and Damhusåen. **Figure S20** shows the variance partition explained by each monitored for the top 25 growing species in each WWTP.

Temperature was a major explanatory variable explaining up to 40% of the variance for some species in both Aalborg West and Damhusåen. As expected, it showed a repeatable seasonal pattern for both WWTPs. Some of the influent characteristics, such as the soluble COD (as a proxy for readily available organic matter), insoluble phosphorus or nitrogen (without ammonia) were also important (explaining ~18-40% of the variance for some species). However, those parameters appear to be WWTP-specific and display a random variation during the year (**Figure S18, supplementary file S2-S3-S4**). Some performance parameters, e.g., SVI in Damhusåen and effluent nitrate concentration (both WWTP), were associated to the variability of species. Both SVI (Damhusåen) and effluent nitrate concentration were seasonal (**supplementary file S3-S4**). However, these parameters are typically considered a response-variables from bacterial activity and whether the change in those parameters can affect back the microbial community is unknown. Nevertheless, for all growing species, between 18 – 90% of the variance could not be explained by any of the monitored parameters. Currently, monitoring parameters are chosen to evaluate the process performance, aid with process control and comply with legislation. However, these bulk measurements appear inconclusive to evaluate their impact on species dynamics, especially to explain systematic variations. A harmonisation of measured parameters as well as detailed studies identifying the most relevant measurements may improve the understanding of microbial dynamics in engineered systems.

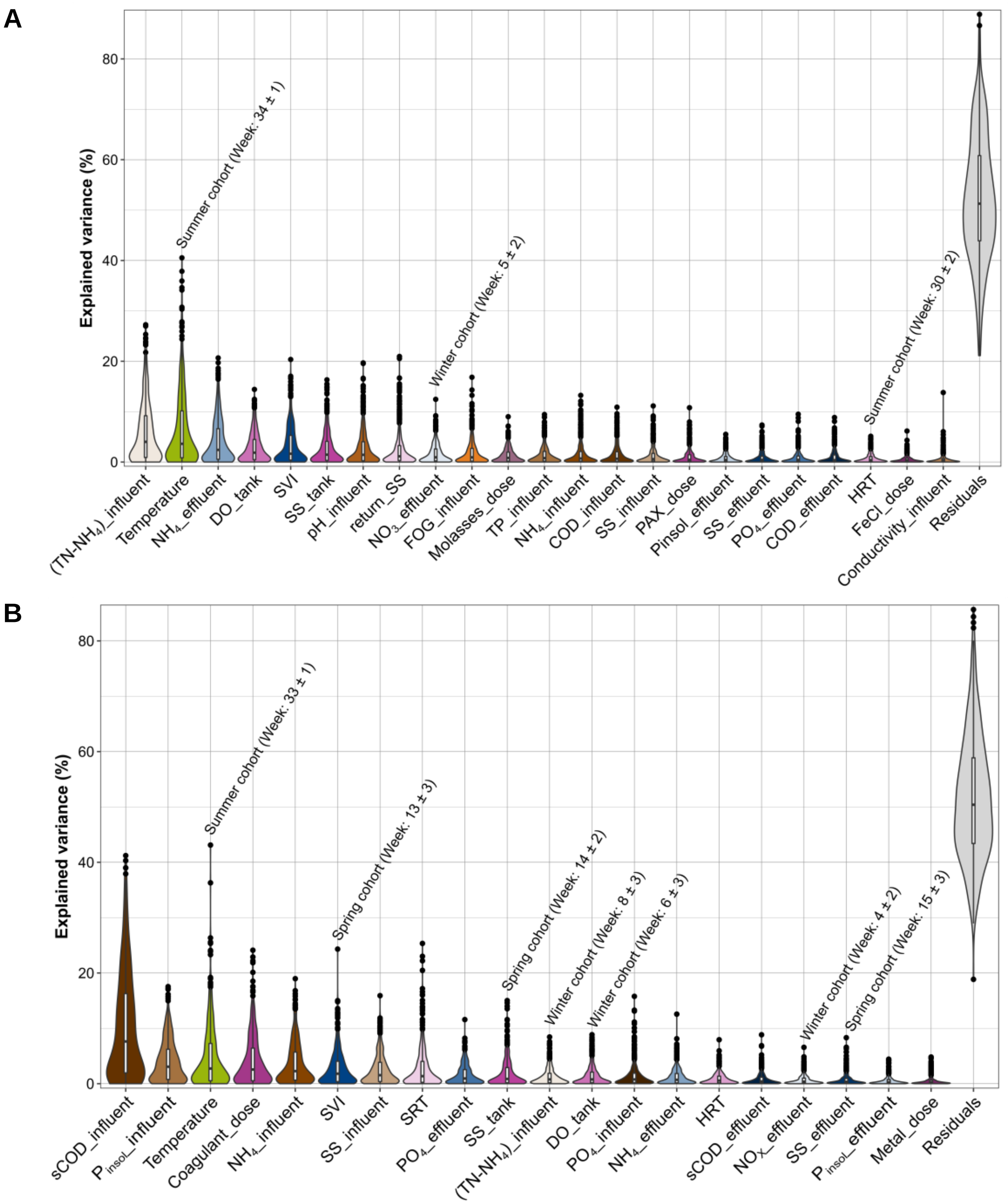

Figure S19. Explained variance of the monitoring parameters for all growing species in each WWTP. (A) Aalborg West. (B) Damhusåen. Violin plots show the distribution of variance explained by each parameter depending on each growing species. The residual fraction (Residuals) represents the variance not explained by any of the monitoring parameters.

Supplementary material: *Microbial communities across activated sludge plants show recurring species-level seasonal patterns*

A

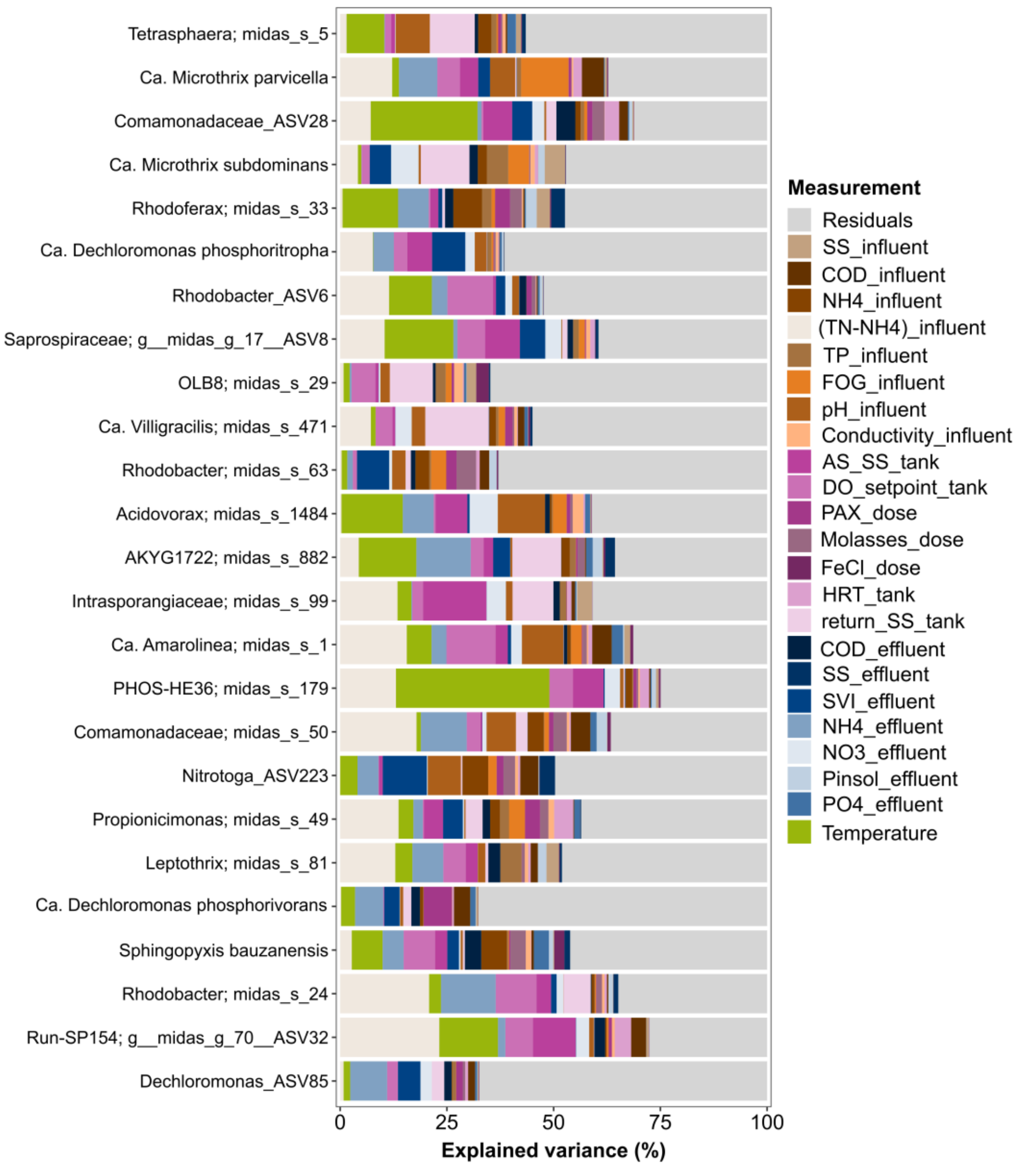

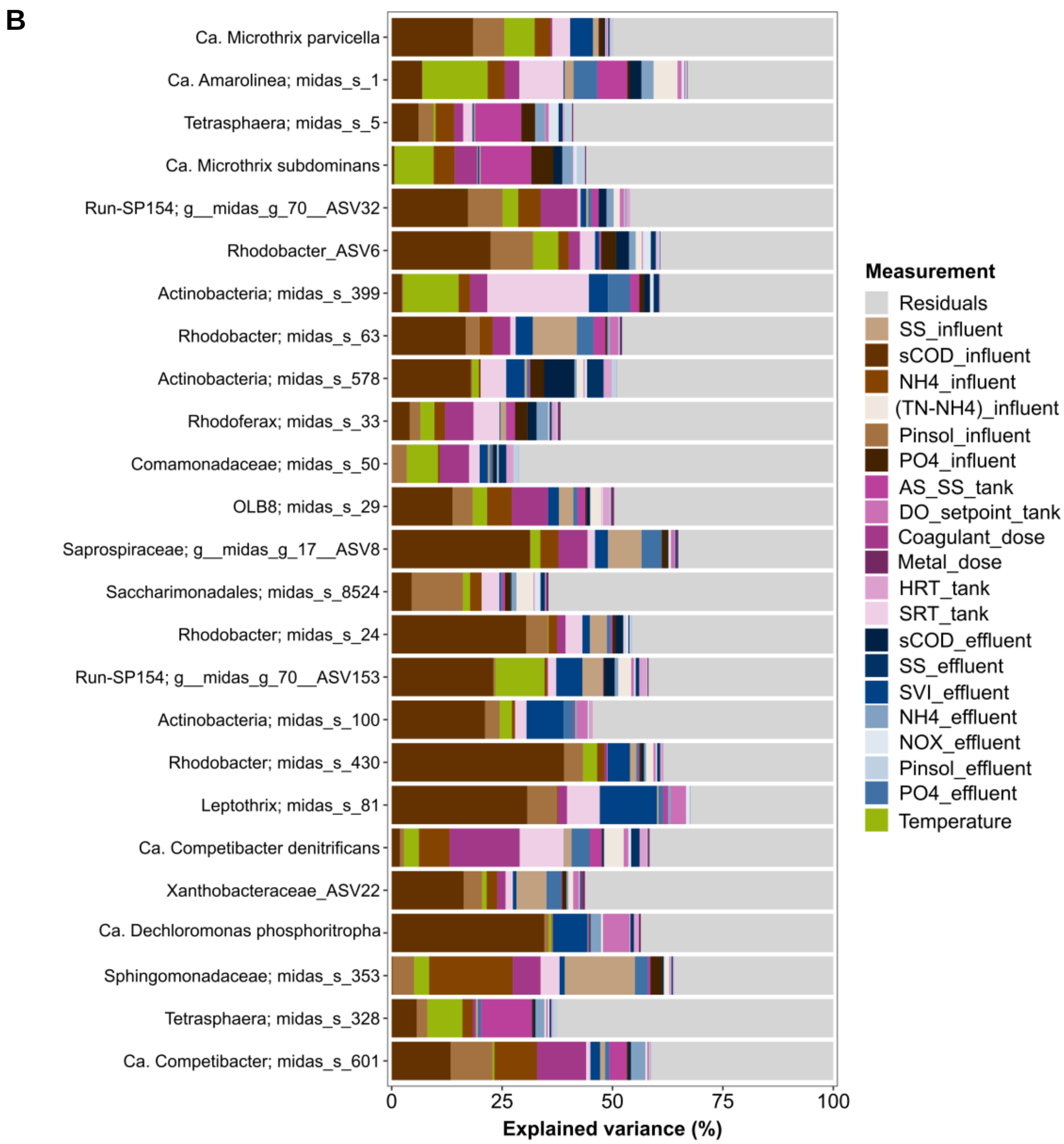

Figure S20. Explained variance of the monitoring parameters for individual species in each WWTP. (A) top 25 growing bacteria in Aalborg West. (B) top 25 growing bacteria in Damhusåen.

References

Dawson, R.N. and Murphy, K.L., 1972. The temperature dependency of biological denitrification. *Water Research*, *6*(1), pp.71-83.

Metcalf & Eddy, Tchobanoglous G, Stensel HD, Tsuchihashi R, Burton F. Wastewater Engineering: Treatment and Resource Recovery, 5th Ed. 2014. McGraw-Hill Education, New York, USA.

Oh, J. and Silverstein, J., 1999. Oxygen inhibition of activated sludge denitrification. *Water Research*, 33(8), pp.1925-1937.

Hoffman, G.E. and Schadt, E.E., 2016. variancePartition: interpreting drivers of variation in complex gene expression studies. *BMC bioinformatics*, *17*(1), pp.1-13.
